# Cell survival following direct executioner-caspase activation

**DOI:** 10.1073/pnas.2216531120

**Published:** 2023-01-20

**Authors:** Maddalena Nano, James A. Mondo, Jacob Harwood, Varuzhan Balasanyan, Denise J. Montell

**Affiliations:** ^a^Molecular, Cellular, and Developmental Biology Department, University of California, Santa Barbara, CA 93106; ^b^Neuroscience Research Institute, University of California, Santa Barbara, CA 93106

**Keywords:** anastasis, apoptosis, effector caspase, recovery, predictive power

## Abstract

Executioner-caspase activation has been considered a point-of-no-return in apoptosis. However, numerous studies report survival from caspase activation after treatment with drugs or radiation. An open question is whether cells can recover from direct caspase activation without pro-survival stress responses induced by drugs. To address this question, we engineered a HeLa cell line to express caspase-3 inducibly and combined it with a quantitative caspase activity reporter. While high caspase activity levels killed all cells and very low levels allowed all cells to live, doses of caspase activity sufficient to kill 15 to 30% of cells nevertheless allowed 70 to 85% to survive. At these doses, neither the rate, nor the peak level, nor the total amount of caspase activity could accurately predict cell death versus survival. Thus, cells can survive direct executioner-caspase activation, and variations in cellular state modify the outcome of potentially lethal caspase activity. Such heterogeneities may underlie incomplete tumor cell killing in response to apoptosis-inducing cancer treatments.

## Significance

Recent work in a variety of cells and organisms shows that cells can recover from the brink of cell death. This property enhances tissue repair after injury but is also co-opted by cancer cells to survive chemo- and radiation therapies. By combining sophisticated tools to deliver and measure precise doses of caspase-3 activity directly in living cells, we address two key questions regarding recovery from apoptotic induction. Can cells recover from direct activation of executioner-caspase, or is survival a consequence of drug-induced stress responses that occur together with death signaling? And, to what extent do caspase signaling levels and/or dynamics determine cell fate? Here, we report the extent and limitations of caspase activity in determining cell fate.

Apoptosis, a form of programmed cell death, is required for tissue homeostasis (1). The balance between cell survival, proliferation, and death ensures proper development and health of multicellular organisms. Excessive cell death occurs in degenerative diseases, ischemia, and immune disorders (2). Survival of abnormal cells can directly support cancer development and recurrence (3, 4). Cancer cells can also use apoptosis as a weapon, by triggering T cell death (5).

Apoptosis is initiated by death-inducing ligands or by intrinsic damage (6–9). In either case, apoptosis culminates in the activation of cysteine-aspartic proteases called caspases, which dismantle the cell by cleaving numerous substrates (10–12). While many forms of death are reversible even at late stages (13–15), apoptosis was considered irreversible after caspase activation and mitochondrial outer membrane permeabilization (MOMP) (16–18).

Recent results demonstrate that cells can survive apoptotic stimuli in a process that has been called fractional killing (19), *anastasis* (20), or failed apoptosis (21). Anastasis is defined as survival from a transient stimulus that activates executioner-caspase and would be lethal if sustained. It has been observed in multiple cell types in vitro (21–28) and in vivo—in mouse neurons (29) and in multiple *Drosophila* tissues (30–32). Molecular mechanisms supporting cell survival include proteins such as Snail, Akt1, and dCIZ1 (26, 31).

Small molecules commonly used to induce apoptosis are pleiotropic. For example, staurosporine is a broad-spectrum kinase inhibitor, and ethanol induces many kinds of cellular stress and damage. Thus, a critical question is whether cells can recover from direct executioner-caspase activation in the absence of concomitant pro-survival stress responses. To address this question, we engineered a system to study the effects of direct effector caspase-3 activation. Using this system, we found that cells can survive direct, transient caspase-3 activation. As expected, high doses of caspase uniformly led to cell death and low doses to cell survival, while intermediate caspase activity was compatible with either outcome. We conclude that heterogeneities in cell states determine the outcome of exposure to levels of caspase activity that are potentially lethal. We identify cellular stress as one factor that influences the response to intermediate caspase activity. Taken together, these results indicate that, in addition to mechanisms such as genetic drug resistance, anastasis may be a source of tumor repopulating cells and recurrence after apoptosis-inducing cancer treatments.

## Results

### Precise Control of Apoptotic Stimulation with Photoactivatable CaspaseLOV.

**Fig. 1.**
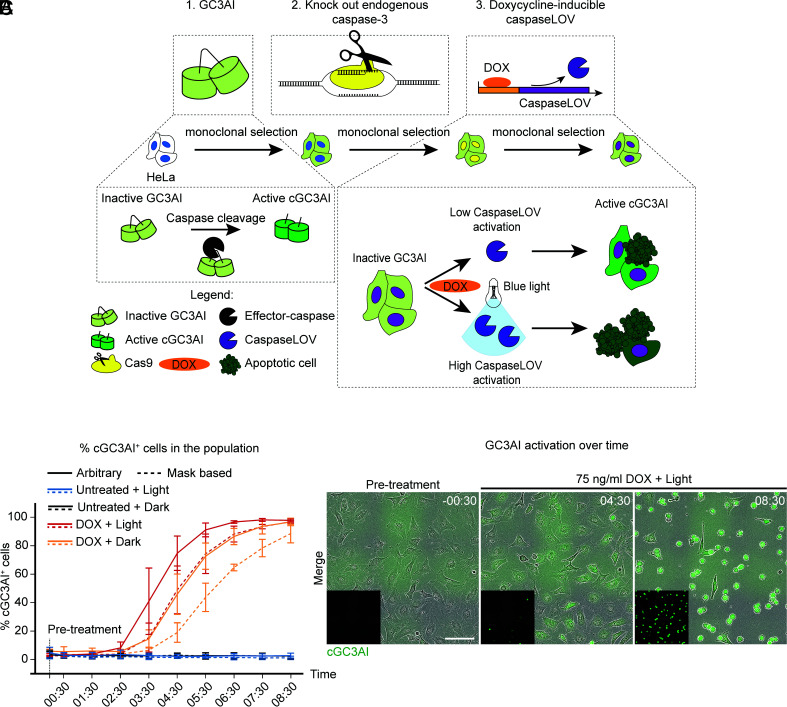
Precise control of apoptotic stimulation with photoactivatable CaspaseLOV. (*A*) Schematic representation of how we generated caspaseLOV cells. The caspase sensor GC3AI was stably introduced in HeLa cells by lentiviral transduction. Endogenous caspase-3 was knocked out by CRISPR-Cas9. Cells were further transduced with DOX-inducible caspaseLOV. Cells underwent monoclonal selection and validation after each manipulation. GC3AI is cleaved and activated by effector caspases. DOX induces caspaseLOV expression and GC3AI activation. Blue light illumination enhances caspaseLOV activity. (*B*) Line chart indicating the percentage of GC3AI^+^ cells over time in caspaseLOV expressing cells in response to different treatments. GC3AI is inactive in the absence of DOX, it is maximal when 75 ng/mL DOX treatment is combined with illumination and it is delayed in the dark. Dotted lines: threshold-based identification of GC3AI positivity and manual counting. Solid lines: arbitrary (eye-based) estimation of GC3AI positivity and manual counting. Automated quantifications provided a rapid overview of cGC3AI increase over time. Manual counting confirmed the automated analysis and allowed quantification of both cGC3AI^+^ and cGC3AI^−^ cells. By 8 h 30 min post-treatment 98% of DOX-treated, illuminated cells and 97% of non-illuminated cells are cGC3AI^+^ (n = 412/420 illuminated cells, n = 497/515 non-illuminated cells, n = 3 independent experiments), compared to ≤4% of untreated controls (n = 11/464 cGC3AI^+^ illuminated cells, n = 9/459 cGC3AI^+^ non-illuminated cells; n = 3 independent experiments). Time post-treatment is in hh:mm. Error bars = SD. (*C*) Images from a timelapse series of HeLa cells stably encoding the effector caspase sensor GC3AI (green) and DOX-inducible caspaseLOV. Cells were treated with 75 ng/mL DOX and exposed to blue light. Over time, cells undergo sensor activation and shrinkage, indicative of cell death. *Insets* show cGC3AI. (Scale bar, 100 µm). Time is in hh:mm.

To assess whether cells can recover from direct executioner-caspase activation, we generated a stable monoclonal HeLa cell line lacking endogenous effector caspase-3 (Fig. 1*A*). To titrate caspase activity, we expressed a form of caspase-3, CaspaseLOV, that uses the blue light-sensitive light-oxygen-voltage (LOV) domain to cage cleaved and activated caspase (33), under the control of a doxycycline (DOX)-inducible promoter (Fig. 1*A*). To monitor effector caspase activation, we employed the genetically encoded caspase-3 activity sensor GC3AI (GFP-based, caspase-3-like, protease activity indicator), which is locked in the dark state until cleaved by caspases (34) (Fig. 1*A*).

Live cell imaging confirmed GC3AI cleavage upon caspase activation, generating fluorescent GC3AI (cGC3AI) (Fig. 1 *B* and *C*). DOX accelerated caspase activation, and blue light further increased the rate (*SI Appendix*, Fig. S1 *A*–*F*). 75 ng/mL DOX was the lowest concentration inducing consistent GC3AI activation with reliable separation between dark and illuminated cells (*SI Appendix*, Fig. S1*E*). Therefore, we used 75 ng/mL DOX for all subsequent experiments.

Although light accelerated the appearance of cGC3AI^+^ cells, both dark and illuminated samples reached about ~97% cGC3AI^+^ cells by 8 h 30 min post-DOX treatment, compared to ≤4% of untreated controls. GC3AI activation was followed by cell death, as judged by cell rounding, shrinkage, and detachment from the substrate (Fig. 1*C*). CaspaseLOV expression increased from 2 h post-DOX, and cleavage of a validated downstream target of endogenous caspase (poly (ADP-ribose) polymerase, PARP) (35) increased from 5 h post-DOX (*SI Appendix*, Fig. S2 *A*–*C*). We conclude that DOX-inducible caspaseLOV efficiently induces cell death through direct effector caspase activation.

### Cells can Survive Direct Caspase Activation.

**Fig. 2.**
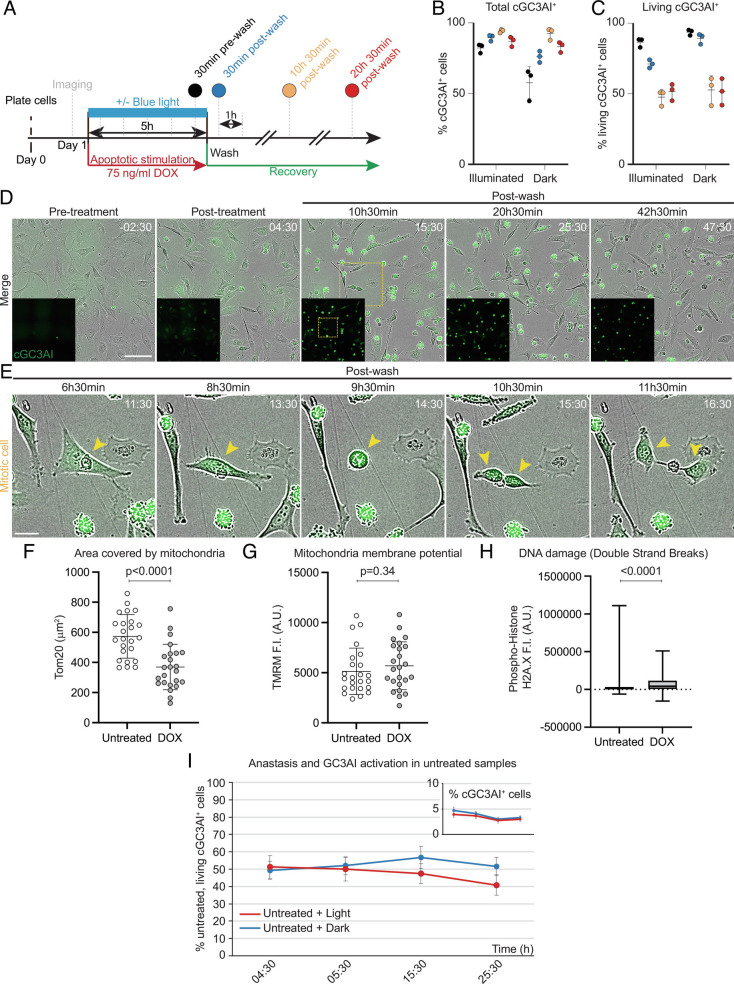
Cells can survive direct caspase activation. (*A*) Experimental design of recovery experiment. Apoptotic stimulation was carried out for 5 h with 75 ng/mL DOX, ±blue light illumination, and was then removed to allow cells to recover. After a pre-treatment acquisition, the sample was imaged hourly, starting 30 min post-treatment. (*B*, *C*) Dot plots indicating the percentage of total cGC3AI^+^ cells (*B*) and living cGC3AI^+^ cells (*C*) at different times after apoptotic stimulation (75 ng/mL DOX ± blue light illumination). (*B*) By the end of 5 h treatment (30 min pre-wash, black), 82% of illuminated cells activated GC3AI (n = 2,421/2,954 cells). The fraction of cGC3AI^+^ cells increased over time, reaching 94% 10 h 30 min post-wash (n = 3,104/3,296 cells, yellow). In the dark, 58% of cells activated GC3AI by 5 h (n = 837/1,437 cells, black) and 92% by 10 h 30 min post-treatment (n = 1,438/1,556 cells, yellow). (*C*) 51% of cGC3AI^+^ cells survive up to 20 h 30 min post-wash (n = 1,615/3,169 illuminated cGC3AI^+^ cells and 706/1,390 non-illuminated cGC3AI^+^ cells, red). Error bars = SD. Each dot represents one experiment (n = 3). (*D*) Images from a timelapse series of caspaseLOV-overexpressing cells during a recovery experiment. GC3AI is in green and in the *Insets*. (Scale bar, 100 µm). Time post-treatment is in hh:mm. Yellow square outlines the mitotic cell magnified in *E*. Related to *SI Appendix*, Fig. S2*G*. (*E*) cGC3AI^+^ cell undergoing mitosis. (Scale bar, 25 µm). (*F*) Quantifications of mitochondrial area (*F*), mitochondria activity (*G*), and DNA damage (*H*) in cGC3AI^-^ untreated cells and cGC3AI^+^ anastatic cells. Fluorescence intensity (F.I.) is shown in Arbitrary Units (A.U.). Statistical significance: unpaired *t* test (*F*) and Mann–Whitney test (*G*, *H*). Error bars = SD. (*I*) Line chart indicating the frequency of spontaneous anastasis in untreated cells exposed to light (red) or kept in the dark (blue). The frequency is robust and similar to what observed for DOX-treated cells 10 to 20 h after treatment (Fig. 2*C*). *Inset* shows the percentage of untreated cells experiencing spontaneous caspase activation, for the same time points shown in the main graph. Error bars = SD. More than 2,000 total cells and more than 75 cGC3AI^+^ cells were quantified for every time point. n = 3 independent experiments.

To address whether cells can undergo anastasis following direct caspase activation, we transiently exposed caspaseLOV cells to DOX, plus or minus blue light illumination. After 5 h, we removed DOX and monitored cell survival for at least 20 h (Fig. 2*A*). After 5 h illumination, 82% of cells activated GC3AI, and the fraction of cGC3AI^+^ cells continued to rise even after the wash, reaching 94% at 10 h 30 min post-wash (Fig. 2*B*). In the dark, caspaseLOV activation was slower. 58% of cells activated GC3AI by 5 h, and 92% by 10 h 30 min post-wash (Fig. 2*B*). Consistent with the continued increase in cGC3AI^+^ cells over time, caspase expression persisted after DOX washout (*SI Appendix*, Fig. S2 *D* and *E*).

Remarkably, 20 h post-treatment, 51% of cells activating GC3AI still exhibited normal morphology, consistent with cell survival after direct caspase activation (Fig. 2*C*). Illumination, including light used for imaging, caused more cells to die (*SI Appendix*, Fig. S2*F*).

Some cGC3AI^+^ surviving cells underwent mitosis (Fig. 2 *D* and *E*) and thus were neither quiescent nor senescent and contributed to repopulating the well (*SI Appendix*, Fig. S2*G* and Movies S1 and S2). cGC3AI was detectable in living cells at even later time points (*SI Appendix*, Fig. S2*G*). Surviving cells contained fewer mitochondria (Fig. 2*F*) but with normal mitochondrial potential (Fig. 2*G*), suggesting they had specifically lost non-functional mitochondria. Surviving cells had a small but significant increase in DNA double strand breaks (Fig. 2*H*), but did not detectably expose phosphatidylserine, which only labeled dead cells (*SI Appendix*, Fig. S2*H*). Surviving cells were not simply completely resistant to death because the dying fraction increased with increasing concentration or duration of DOX treatment (*SI Appendix*, Fig. S2*I*). Furthermore, the population that died could be rescued by the pan-caspase inhibitor Q-VD-Oph (Q-VD), confirming that the death we observed was caspase-dependent (*SI Appendix*, Fig. S2*J*). We conclude that direct effector caspase activity sufficient to kill many cells is nevertheless compatible with survival of others.

### Anastasis Frequencies Are Similar in Cells with Induced or Spontaneous Caspase Activity.

In the absence of DOX, cells did not express caspaseLOV (*SI Appendix*, Fig. S2 *A* and *B*). Although they lacked caspase-3, they were nevertheless competent to undergo apoptosis in response to staurosporine (*SI Appendix*, Fig. S2*K*), presumably mediated by caspase-7. In the absence of DOX, spontaneous GC3AI activation was rare (<5%) in both dark and illuminated cells (*SI Appendix*, Fig. S1*A* and Fig. 2*I*). Remarkably, anastasis occurred in ~50% of untreated cells that spontaneously activated cGC3AI^+^ (Fig. 2*I*), similar to the frequency for DOX-treated cells (Fig. 2*C*). We conclude that the probability of anastasis is similar whether caspase is activated spontaneously or artificially and that heterogeneities in the cell population determine life or death in response to potentially lethal caspase levels.

### Surviving Sister Cells Can Have Higher Caspase Levels than Their Dying Siblings.

**Fig. 3.**
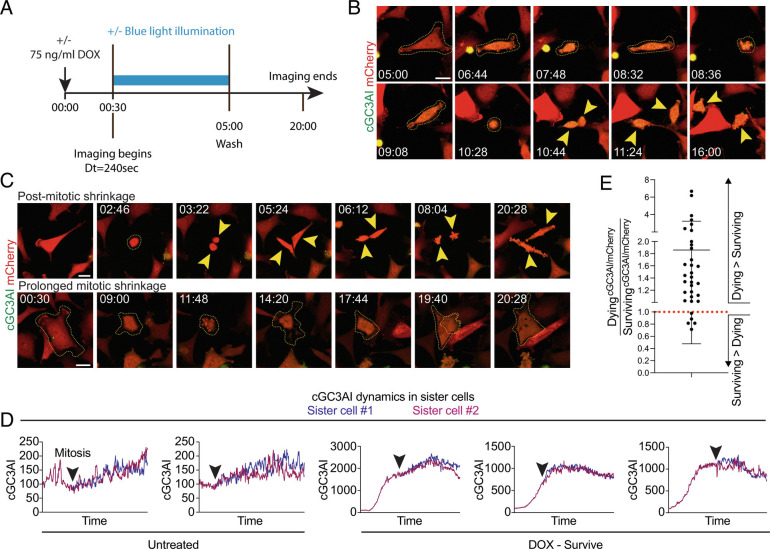
Surviving sister cells can have higher caspase levels than their dying siblings. (*A*) Experimental design of long-term live imaging experiment. After 30 min of apoptotic stimulation (75 ng/mL DOX), cells were imaged ±blue laser illumination every ~240 s (see *Methods*). Controls were not stimulated with DOX but were illuminated to control for phototoxicity. (*B*) Stills of a caspaseLOV cell undergoing mitosis after DOX treatment. GC3AI is in green. Stable mCherry expression (red) was used to normalize cGC3AI fluorescence. Yellow dotted lines outline the parental cell boundaries. Yellow arrowheads indicate daughter cells. The cell undergoes several brief shrinkage events. Time post-treatment is in hh:mm. Time 05:00 is the first acquisition post-washout. (Scale bar, 25 µm). (*C*) Representative examples of shrinkage. Time post-treatment is in hh:mm. (Scale bar, 25 µm). *Top* panels show daughter cells that undergo shrinkage after cell division. Yellow dotted lines outline the parental cell. Yellow arrowheads indicate daughter cells. *Bottom* panels show a parental cell undergoing a first round of abortive mitosis (notice the darker mCherry signal in the center of the cell, where the metaphase plate is). The cell blebs and attempts mitosis again, this time generating two superimposed cells that fuse in a single daughter. Yellow dotted lines outline cell boundaries. (*D*) Line graphs of representative cGC3AI dynamics in surviving sister cells (expressed as cGC3AI IntDen/mCherry IntDen and normalized). Note that y axes are not equivalent. Time is from beginning to end of imaging (~20 h). Black arrowheads indicate mitosis. (*E*) Dot plot chart showing the cGC3AI/mCherry ratio in dying versus surviving sister cells. A ratio greater than 1 (red dotted line) represents higher caspase activity in the dying sister, a ratio of 1 represents equal activity between sisters, and a ratio lower than 1 represents higher caspase activity in the surviving sister. In 5/33 couples of siblings analyzed, the surviving sister exhibited higher levels of caspase than its dying sibling.

To address whether some feature of caspase dynamics, rather than dose, at the single-cell level determined apoptosis vs. survival, we conducted long-term live imaging. We included an mCherry marker to normalize the cGC3AI signal, and this cell line turned out to be less sensitive to DOX (~21% survival after 24 h of DOX without washout, vs. ~1.6% in parental cells). However, the cell line responded well to apoptotic stimulation, since ~24% of the population died after DOX treatment followed by washout (*SI Appendix*, Table S1) and most cells activated GC3AI over time. Imaging began 30 min post-DOX and every 4 min thereafter, plus or minus blue light. After 5 h of DOX, cells were washed and imaged overnight (Fig. 3*A*). DOX plus blue laser stimulation promoted cell death (*SI Appendix*, Table S1, see *Methods*).

The fraction of mitotic cells was similar between treated and untreated samples (42% vs. 44%, respectively), confirming that anastatic cells could proliferate (*SI Appendix*, Table S1, Fig. 3 *B* and *C*, and Movies S1 and S2). >98% of daughters from the same mitosis shared the same fate (*SI Appendix*, Table S1) and exhibited similar cGC3AI dynamics (Fig. 3*D* shows representative examples). However, we found 33 sister cell pairs with opposite fates, and in 5/33 cases, the surviving sister exhibited a higher level of caspase than its dying sibling (Fig. 3*E*). These results show that the difference between death and survival does not only depend on a specific caspase activity threshold, even in closely related cells, and suggest that additional factors contribute to the outcome when cells experience intermediate caspase activation.

### Caspase Activity Dynamics Does Not Fully Predict Cell Fate.

**Fig. 4.**
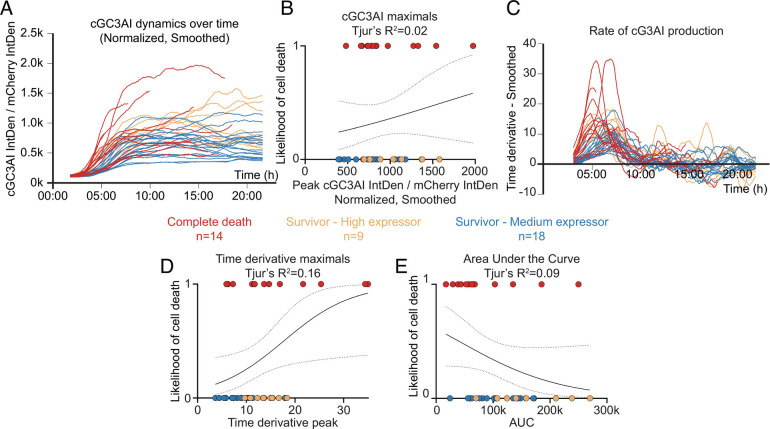
Cell survival and cell death choices can occur independently of caspase dynamics. (*A-E*) Relationship between caspase activity and commitment to cell death. Data points are color-coded by fate or according to the level of total GC3AI (tGC3AI) measured by immunostaining (red = dying cells; orange = surviving cells with high tGC3AI; blue = surviving cells with average levels of tGC3AI). n = number of cells analyzed for each condition (from three independent experiments). (*A*) Line graph showing the dynamics of caspase activation over time in dying vs. surviving cells. Dynamics were obtained as percentage fold change of the ratio cGC3AI/mCherry over time after normalization. Before representation, data were smoothed using a moving average of 20. Smoothed data were used for further analysis. Curves pre-smoothing are shown in *SI Appendix*, Fig. S3 *B* and *C*. (*B*) Logistic regression between the maximum fold change recorded for cGC3AI (denoted “maximal” or “peak”) and the likelihood of cell death (0 = alive, 1 = dead) related to *SI Appendix*, Figs. S3*D* and S6*A*. (*C*) Line graph showing the smoothed time derivative of curves in *A*. Derivatives pre-smoothing are shown in *SI Appendix*, Fig. S3*E*. (*D*) Logistic regression between the maximum of the time derivative (denoted maximal or peak) and the likelihood of cell death (0 = alive, 1 = dead) related to *SI Appendix*, Figs. S3*F* and S6*B*. (*E*) Logistic regression between the area under the curve (AUC) of curves in *A* and the likelihood of cell death (0 = alive, 1 = dead) related to *SI Appendix*, Fig. S6*C*. Dotted lines represent the 95% confidence interval (C.I.). Tjur’s R^2^ evaluates the goodness of fit.

To further probe the relationship between caspase activity, GC3AI cleavage, and commitment to apoptosis, we carried out gentle live imaging using low laser power. To control for possible differences in levels of total GC3AI (tGC3AI), we used correlative live and fixed confocal microscopy. We measured tGC3AI and selected surviving cells with average (medium expressors) or high expression levels (high expressors, see *Methods*). We measured the change in caspase activity over time and compared cells that lived to those that died (Fig. 4*A* and *SI Appendix*, Fig. S3 *A*–*C*, and Movies S3 and S4). To address how well caspase-3 activity predicted death, we ran a logistic regression analysis (*SI Appendix*, Fig. S4 *A*–*C*). Cells that survived were assigned a value of 0 and those that died a value of 1. We plotted these fates against cGC3AI fluorescence. If all dying cells had high and all surviving cells had low cG3AI levels, 0s and 1s would segregate completely, indicating that cGC3AI is a perfect predictor of fate (*SI Appendix*, Fig. S4*A*). By contrast, we found that the fold change in caspase activity predicted the fates of only 2% of cells (Fig. 4*B*), or 11% when the analysis was restricted to medium expressors (*SI Appendix*, Fig. S3*D*).

Roux et al. (2015) reported that the maximal rate of caspase-8 activation predicts cell death with >80% accuracy in cells treated with the death-inducing ligand Trail. We also found that the rate of caspase-3 activation predicted cell fate better than maximal fold change. However, when we plotted the rate at which cGC3AI is produced (Fig. 4*C* and *SI Appendix*, Fig. S3*E*), the predictive power was only 16% (Fig. 4*D*), or 24% when the analysis was restricted to medium expressors (*SI Appendix*, Fig. S3*F*). Dying cells tended to reach GC3AI maximal fold change earlier than surviving cells (*SI Appendix*, Fig. S3*G*), while the time at which the rate of caspase activity peaked was similarly for dying and surviving cells (*SI Appendix*, Fig. S3*H*).

To assess whether total accumulated caspase activity accounts for apoptotic commitment, we measured the area under the curve (AUC) of cGC3AI in dying and surviving cells. Similar to the maximal rate, the fold change, and the timing of caspase activity, AUC was insufficient to explain cell fate choices (Fig. 4*E*).

This quantitative analysis allowed us to explore correlations between the different caspase dynamics parameters (*SI Appendix*, Fig. S5). Overall, faster, stronger, or longer caspase activation did not necessarily lead to cell death.

Finally, we tested whether any parameter of caspase activity would be strongly predictive of cell fate if we restricted the analysis to the early phase of apoptotic stimulation. However, even when considering only the first 100 time points of imaging (up to 428 min post-treatment), caspase dynamics did not reliably predict survival vs. death (*SI Appendix*, Fig. S6 *A*–*C*). Interestingly, we found that maximal cGC3AI fold change had higher predictive power when considering only its early values (Tjur’s R^2^ = 0.02 vs. 0.15), consistent with faster cGC3AI accumulation in dying cells (Fig. 4*D*). Consistently, we found an inversion in the logistic relationship between cell survival and AUC (compare Fig. 4*E* and *SI Appendix*, Fig. S6*C*). Total AUC was moderately larger in surviving cells (Fig. 4*E*) because they accumulated cGC3AI for longer. However, initial AUC was slightly larger in dying cells (*SI Appendix*, Fig. S6*C*), indicating that they accumulated cGC3AI faster. Taken together, our results demonstrate that direct effector caspase activation is compatible with cell survival. In addition, they show that surviving cells somehow tolerate total levels of caspase activity comparable to those in dying cells.

**Fig. 5.**
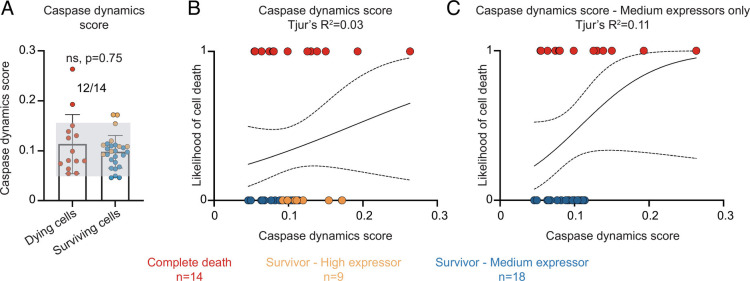
The cell state can determine whether a cell lives or dies. (*A-C*) Caspase dynamic score in dying (red) and surviving cells (orange = surviving cells with high tGC3AI; blue = surviving cells with average levels of tGC3AI). n = number of cells analyzed for each condition (from three independent experiments). (*A*) Dot plot chart and histogram showing the death score of dying and surviving cells. Error bars = SD. Lack of statistical significance (*P *= 0.75) was assessed with Mann–Whitney test. Gray shading helps visualize dying and surviving cells with overlapping death scores. The numbers on top indicate the fraction of dying cells with death scores overlapping with surviving cells. (*B* and *C*) Logistic regression between the death score and the likelihood of cell death (0 = alive, 1 = dead) considering all surviving cells (*B*) or medium expressors only (*C*). Dotted lines represent the 95% C.I.. Tjur’s R^2^ evaluates the goodness of fit.

To test whether a combination of caspase dynamics parameters might better predict cell fate than any individual feature, we combined all parameters of caspase dynamics into a single numerical factor that we named “caspase dynamics score” (see  *Methods*). We found that, when combined, the time derivative, the cGC3AI maximal, and the AUC performed worse than the individual parameters in predicting whether cells lived or died (Fig. 5 *A*–*C*). When the score was calculated only on the initial dynamics of cGC3AI, its predictive power did not exceed 30% (*SI Appendix*, Fig. S6 *D*–*F*). These results again suggest that additional factors influence life vs. death fates in response to intermediate levels of caspase activity (*SI Appendix*, Fig. S6*G*).

### Stress Increases the Predictive Power of the Rate of Caspase Activation.

In considering which aspects of cell state could affect the predictive power of caspase activation, we noticed that stressful imaging conditions increased cell death frequency, suggesting that stress might be a factor that tips the balance between life and death at intermediate caspase levels. To manipulate stress and apoptotic stimulation simultaneously, we turned our attention to the more sensitive caspaseLOV cell line that did not express mCherry (Figs. 1 and 2 and *SI Appendix*, Figs. S1 and S2 *A*–*J*) and delivered phototoxic stress.

**Fig. 6.**
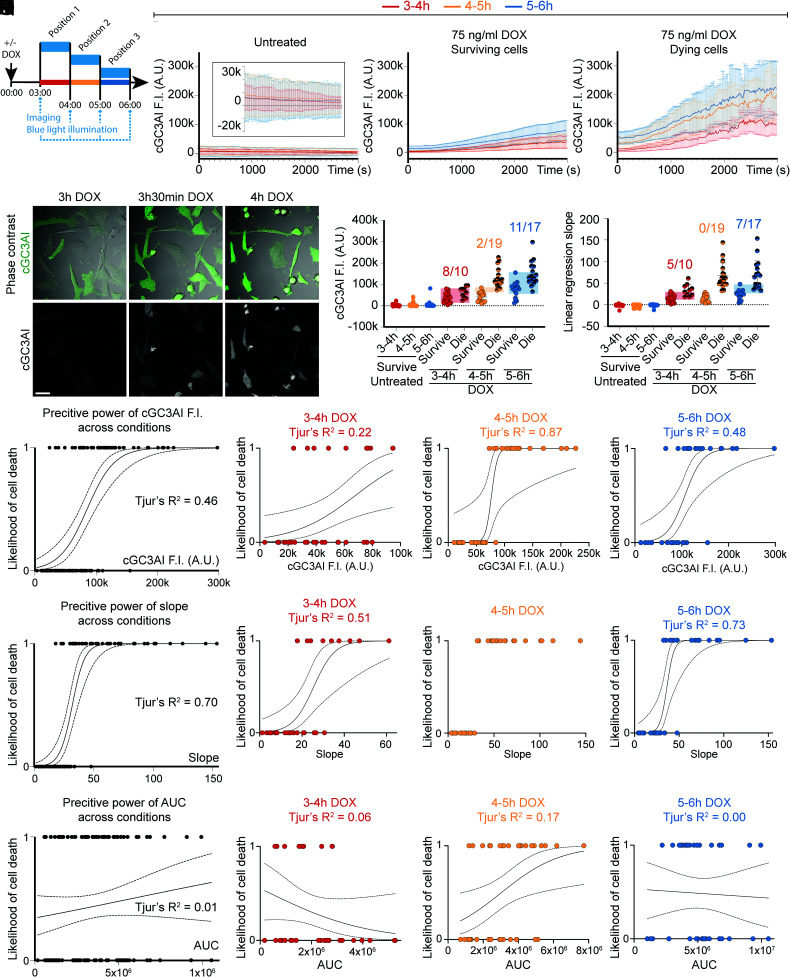
Stress increases the predictive power of the rate of caspase activation. (*A*) Experimental design. After 3 h of apoptotic stimulation (75 ng/mL DOX), the sample was imaged and stimulated with blue light at ~20 s interval. Three positions were imaged over three consecutive hours: position 1 (3 to 4 h DOX, red, n = 33 cells), position 2 (4 to 5 h DOX, orange, n = 37 cells), and position 3 (5 to 6 h DOX, blue, n = 35 cells). Controls were imaged in the same way but were not stimulated with DOX. Data are from three independent experiments. (*B*–*D*) Line graphs of GC3AI F.I. in A.U. in untreated (*B*) and DOX-treated surviving (*C*) and dying (*D*) cells. Data plotted are the average of all cells analyzed and are relative to the first (red), second (orange), or third (blue) hour of imaging. The *Inset* in *B* shows the same data on an expanded y axis. Error bars = SD. (*E*) Stills of DOX-treated HeLa cells expressing caspaseLOV and GC3AI (green in the merged panel and gray). (Scale bar, 33 µm). (*F*–*G*) Dot plots indicating cGC3AI F.I. (*F*) and slope of the linear regression of GC3AI F.I. (*G*). Data are relative to the first (red), second (orange), or third (blue) hour of imaging. Semi-transparent rectangles help visualizing dying and surviving cells with overlapping values. The numbers on top indicate the fraction of dying cells with values overlapping with surviving cells. (*F*) Dot plot showing the last value of GC3AI F.I. measured in untreated and DOX-treated surviving and dying cells. Surviving cells were measured at the last time point imaged, and dying cells were measured at cell death. (*G*) Dot plot showing the slope of linear regressions calculated on GC3AI F.I. (until the end of imaging for surviving cells, until death for dying cells). (*H*–*S*) Logistic regressions between parameters of caspase activity and cell survival. Tjur’s R^2^ evaluates the goodness of fit. Dotted lines represent 95% C.I.. (*H*–*K*) Logistic regression between the last value of GC3AI F.I. and the likelihood of cell death (0 = alive, 1 = dead) for DOX-treated cells in all conditions tested (*H*) and 3 to 4 h (*I*), 4 to 5 h (*J*), or 5 to 6 h (*K*) post-treatment. (*L*–*O*) Logistic regression between the slope of linear regressions of GC3AI F.I. over time and the likelihood of cell death (0 = alive, 1 = dead) in all conditions tested (*L*) and 3 to 4 h (*M*, red), 4 to 5 h (*N*, orange), or 5 to 6 h (*O*, blue) post-treatment. (*P*–*S*) Logistic regression between the AUC of GC3AI F.I. and the likelihood of cell death (0 = alive, 1 = dead) in all conditions tested (*P*) and 3 to 4 h (*Q*, red), 4 to 5 h (*R*, orange), or 5 to 6 h (*S*, blue) post-treatment.

We induced caspaseLOV expression with 75 ng/mL DOX and started live imaging after 3 h, using confocal microscopy to measure cGC3AI fluorescence in single focal planes. Samples were imaged and illuminated with blue light every 20 s for 50 min in a given position (3 to 4 h DOX) (Fig. 6 *A* and *E* and Movie S5) before moving to a different position. Imaging was repeated every hour in a different position for a total of three consecutive hours (3 to 4 h, 4 to 5 h, and 5 to 6 h post-DOX) (Fig. 6*A*). The percentage of cells analyzed for each condition is reported in *SI Appendix*, Table S2.

In control samples, >99% of cells were cGC3AI negative (Fig. 6*B*) and survived until the end of imaging for all time points tested (*SI Appendix*, Table S2). In DOX-treated samples, the percentage of dying cells initially increased over time, peaking 4 to 5 h post-treatment (*SI Appendix*, Table S2). In DOX-treated cells, average cGC3AI fluorescence intensity was higher in cells that died compared to those that survived (Fig. 6 *C* and *D* and *SI Appendix*, Table S3), demonstrating again that high levels of caspase activity are associated with death. We measured cGC3AI fluorescence for all time points (Fig. 6 *B*–*D* and *SI Appendix*, Fig. S7 *A*–*C*, *E*–*G*, *I*–*K*, *M*, *N*, and *P*). However, to evaluate caspase activity predictive power, we stopped our analysis at apoptotic commitment for dying cells (Fig. 6 *F*–*S* and *SI Appendix*, Fig. S7 *D*, *H*, *L*, and *O*), while surviving cells were measured until the end of imaging.

The effect of blue light in this context might simply be to increase caspase activity so that more cells experience lethal levels. Alternatively, the blue light might add a stress that makes it more likely for a cell experiencing an intermediate level of caspase to die. To distinguish between these possibilities, we measured overall levels of caspase activity. For surviving cells, we plotted the last value of cGC3AI fluorescence intensity during the imaging period, and for dying cells, we plotted cGC3AI intensity at the moment of irreversible shrinkage (Fig. 6*F*). We found that final cGC3AI levels could overlap between dying and surviving cells, and many surviving cells reached equal or higher cGC3AI values than many dying cells (Fig. 6*F*), especially at 3 to 4 h but even at 5 to 6 h.

According to the partial overlap observed, logistic regressions demonstrated that cGC3AI fluorescence intensity has limited predictive power for cell fate determination. Across conditions, the model fitted about 46% of the data (Tjur’s R^2^ = 0.46; Fig. 6*H*), with variable performance over time (Fig. 6 *I*–*K*).

To test whether the speed of caspase activation could predict cell fate, we checked how steeply cGC3AI fluorescence intensity rose over time. For dying cells, we stopped our measurement at the moment of shrinkage. When we compared slopes of the linear regression of dying and surviving cells, we found low speeds correlated well with survival until the end of imaging, and higher speeds were associated with cell death (Fig. 6*G*, Fig. 6 *L*–*O*). Logistic regression demonstrated that slopes correctly predicted apoptotic commitment ~70% of the time across conditions (Tjur’s R^2^ = 0.70), leaving ~30% of cell fate choices unaccounted for, although at 4 to 5 h post-treatment speed perfectly predicted fate (Fig. 6*N*).

Finally, we tested the predictive power of total caspase activity over time (Fig. 6 *P*–*S*). However, the AUC of cGC3AI predicted cell fate accurately in only ~1% of the cases across conditions (Fig. 6*P*). These results showed that the same total amount of caspase activity causing cell death was compatible with survival when occurring slowly over prolonged periods of time.

Overall, we found that the added stress of blue light illumination not only increased caspase activity but made its rate of activity and final level better predictors of cell fate (*SI Appendix*, Fig. S8*A*). Under stressful conditions, caspase activation rate became a major determinant of cell death.

To test whether a different type of stress could increase the deterministic value of caspase activity, we tested the effect of a low concentration of the proteasome inhibitor MG-132 (*SI Appendix*, Fig. S8*B*). We found that proteotoxic stress increased the separation of caspase activity between dying and surviving cells (*SI Appendix*, Fig. S8*C*), supporting the idea that stress enhances the role of caspase activity in cell fate decisions.

## Discussion

Here, we develop tools to directly activate and measure caspase-3 dynamics in living cells and use them to address two questions: whether cells can survive direct caspase activation and how well effector caspase dynamics predicts cell survival vs. death. Our results show that cells can recover from direct caspase activation and add to the evidence that the apoptotic cascade is not strictly irreversible (25–27, 30, 31, 36–38).

A variety of mechanisms might promote survival after effector caspase activation. One possible mechanism is negative feedback. Consistent with this idea, pro-survival caspase cleavage products have been identified. For example, caspase-3-cleaved RasGAP promotes survival via Akt signaling (39). In hematopoietic cells, caspase-cleaved IRE1 inhibits MOMP, promoting survival (40).

The integrated stress response (ISR) is renowned for promoting survival in response to low levels of stress while killing cells that experience overwhelming or unremitting stress. In recent work, Kalkavan et al. show that cells can also survive MOMP and acquire a persister phenotype in the absence of executioner-caspase activity (38). They show that released cytochrome c promotes translation of the ISR transcription factor ATF4 and causes drug-treated tumor cells to survive. These persister cells display signs of epithelial to mesenchymal transition (EMT) and are more metastatic in mouse cancer models. Similarly, cells that recover from the brink of death after caspase activation, i.e., anastatic cells, also up-regulate EMT-associated genes, increase their motility (26), and show increased metastasis in mouse cancer models (36).

Another example of a caspase cleavage product that promotes survival is the energy sensor AMPK-α1 (41). Caspase-3 removes a nuclear export sequence from the full-length protein, which causes it to accumulate preferentially in the nucleus where it protects cells from DNA damage-induced death. These studies show that many different cells possess mechanisms to rebound from potentially lethal insults. Cancer cells appear to co-opt these normal abilities, which are useful to promote recovery from severe injury, and exploit them to survive chemotherapy and spread.

Overall, effector caspase activity is not only compatible with cell survival but can promote escape from apoptosis via the molecular mechanisms described above and possibly others that are yet to be identified. We frequently observed proliferation in anastatic cells demonstrating that survival from caspase activity does not necessarily lead to senescence. Together with resistant cells and persister cells, the ability of cells to survive executioner-caspase activation may contribute to tumor cell survival from chemo and radiation therapy and subsequent relapse.

The second question we address in this study is how well effector caspase levels and dynamics predict survival vs. death. We show that high levels of caspase activity do kill all cells and low levels allow all cells to survive. This is consistent with a bistable on/off system, where positive feedback ensures all or nothing responses (42–47). However, we also find that at intermediate levels, caspase activity accounts only partly for cell fate in unrelated cells. In sister cells, caspase levels correlate better with cell fate choices, although insufficiently to fully explain cell death. In this intermediate landscape of activity, neither the peak level, nor the peak rate, nor the total level of executioner-caspase strongly predicts whether a cell survives or dies. On average, dying cells experience higher and faster cGC3AI accumulation, while somewhat counterintuitively, surviving cells can experience more total caspase activity, as they persist longer. We observed that a steep rise in caspase activity increases the likelihood, but does not necessarily spell, certain death. This is consistent with previous work showing that caspase inhibition is permissive to cell survival (48, 49), and that certain ranges of caspase concentration leave space for a bistable but reversible description of apoptosis (46).

For a bistable system to transition from the “off” to the “on” state, the stimulus delivered needs to surpass a threshold. Interruption of apoptosis could result from a sublethal injury that fails to overcome this threshold (44, 47, 49–52). However, we found that cell death occurred in 15 to 30% of cells experiencing intermediate caspase activation, indicating that the apoptotic stimulus we delivered was sufficient to turn on the apoptotic switch. Therefore, this threshold does not depend only on levels or dynamics of active executioner-caspase. Rather, the cell integrates additional information (53), such as its stress level, to make the critical decision whether to live or die (*SI Appendix*, Fig. S8). In anastasis-permissive conditions, this additional information could come in the form of the status of the ISR, mitochondrial health, viral infection, and nutrient status. Other factors, such as DNA damage, reactive oxygen species (ROS), and cell cycle stage, could also contribute to the uniqueness of single-cell responses. Inability to surpass the apoptotic threshold could also depend on cell-to-cell fluctuations in regulators of the amplitude of the apoptotic switch (49–51) such as IAPs (49), rates of caspase turnover (54–56), and different susceptibility to MOMP (44, 47, 52, 57).

Alternatively, at intermediate levels of caspase activity, apoptosis might not behave as a bistable system. As discussed above, caspase-3 could directly establish negative feedback loops generating pro-survival molecules by cleaving specific substrates (39–41). Alternatively, intermediate levels of caspase-3 activation might only stochastically engage the positive feedback loops required to establish bistability (46). Similar scenarios may occur in vivo, during development, when sublethal levels of caspase control cellular remodeling (58) and cell fate choices (59–63). For instance, ROS have been shown regulate caspase activation during the developmental process of *Drosophila* thorax fusion (64).

Overall, our results show that strong apoptotic stimuli will induce cell death irrespective of the cell state, but milder stimuli will have an outcome that depends on multiple factors. In the context of cancer therapy, the strength of apoptotic stimulus experienced by individual cells depends on many complex factors including proximity to blood vessels, position within the tumor, expression of drug efflux pumps, and cell density to name a few. Exposures will therefore vary in space and time, with some cells receiving a strong stimulus but others possibly receiving just enough stimulation for their preexisting state to play a major role in their fate. As a 1 cm^3^ tumor is estimated to contain 10^9^ cells, even a relatively small fraction can represent a physiologically meaningful population.

## Material and Methods

### Cell Culture and Maintenance.

Cells were grown in Dulbecco’s Modified Eagle Medium, high glucose, GlutaMAX™ Supplement, pyruvate (Gibco #10-569-044), 10% heat-inactivated fetal bovine serum (Sigma-Aldrich #F4135), 1 μg/mL puromycin (Gibco #A1113803). All cells were maintained at 37 °C with 5% CO_2_ and 90% humidity. Cells were not tested for mycoplasma contamination.

### Generation of CaspaseLOV Cells.

Genetic manipulations to generate caspaseLOV cells are described in the *SI Appendix, Material and Methods*. Briefly, HeLa cells (ATCC® CCL-2™) were stably transduced with pCDH_GC3AI (34). A single clone obtained by serial dilution was modified by CRISPR-Cas9 (*SI Appendix*, Table S4) to remove the endogenous gene encoding executioner-caspases-3. After monoclonal selection and validation, cells were transduced with a DOX-inducible lentiviral plasmid encoding CaspaseLOV (33). When indicated, caspaseLOV cells were further transduced with pCDH-CMV-mCherry-T2A-Puro and sorted for mCherry positivity using a Sony SH800 Cell Sorter. All primers and plasmids used are listed in *SI Appendix*, Tables S5 and S6.

### DOX Treatment and Blue Light Illumination.

Unless otherwise specified, doxycycline hydrochloride (Fisher Scientific #AAJ67043AD) was added to growth media to a final concentration of 75 ng/mL. Blue light illumination for western blotting, IncuCyte imaging, and short-term confocal imaging is described in the *SI Appendix, Material and Methods*.

### IncuCyte Imaging.

Cells were plated in 24-well cell culture plates at 33,000 cells/well (Genesee Scientific #25-107) the day before the experiment. Cells were imaged hourly on an IncuCyte Zoom (Essen BioScience). Detailed experimental procedures are reported in the *SI Appendix, Material and Methods*.

### Western Blotting.

For caspaseLOV activation dynamics (*SI Appendix*, Fig. S2 *A*–*C*), 500,000 to 1,000,000 cells were treated ±DOX in the presence of blue light illumination and samples were collected at the indicated times post-treatment. For caspaseLOV shutdown dynamics (*SI Appendix*, Fig. S2 *D* and *E*), 300,000 cells were treated 5 h ±DOX. Cells were washed and allowed to recover in fresh media for the indicated time before collection by trypsinization and centrifugation (RT, 2,000 rpm, 5 min). Sample processing and blot quantification are described in the *SI Appendix, Material and Methods*.

### Immunofluorescence.

Samples were fixed for 10 min at 37 °C in 4% paraformaldehyde (Electron Microscopy Sciences #15711), 4% sucrose (Sigma #84097) in PBS (Gibco #20-012-050). Permeabilization, washes, blocking, and antibodies incubation are described in the *SI Appendix, Material and Methods*. Antibodies are listed in *SI Appendix*, Table S7.

### Mitochondria Abundance, Activity, DNA Damage, and Annexin V Assays.

Experimental and quantitative approaches are described in the *SI Appendix, Material and Methods*.

### Long-Term Confocal Imaging.

150,000 caspaseLOV cells expressing mCherry were plated the day before the experiment on a glass-bottom 35 mm Dish (Mattek #NC9903355). 30 min before imaging, cells were switched into 2 mL of imaging media (*SI Appendix, Material and Methods*), ±75 ng/mL DOX. The sample was imaged for ~4 h 30 min every 4 min. 5 h post-treatment, the media was replaced with 3 mL of fresh media (without DOX). The sample was imaged overnight (~15 h 30 min). The 458 nm laser line was used to deliver blue light illumination during the first 4 h 30 min of imaging (at 0%, 5%, and 10% laser power; see *SI Appendix, Material and Methods*). Controls were imaged using the 458 nm laser line at 10% laser power.

Illumination with 0%, 5%, and 10% laser power gave similar results. The results were therefore pooled for presentation in Fig. 3 and *SI Appendix*, Table S1. *SI Appendix*, Table S8 breaks down the data presented in *SI Appendix*, Table S1 according to the 458 nm laser power used. Detailed imaging and quantification protocols are reported in the *SI Appendix, Material and Methods*.

### Correlative Live and Fixed Confocal Microscopy.

After validating our immunofluorescence approach (*SI Appendix, Material and Methods*), we carried out correlative live and fixed confocal microscopy (n = 3 independent experiments). Sample preparation and treatment were carried out as described in the section “*Long-term Confocal Imaging*”, with the only difference that we plated 100,000 cells. Detailed imaging procedures and quantitative workflow are reported in the *SI Appendix, Material and Methods*. At the end of live imaging, the live sample was acquired once more and was then immediately fixed and stained as described in the “*Immunofluorescence*” section of the methods, using antibodies anti-GFP and anti-cleaved caspase-3, detected with a far red and a blue secondary antibody, respectively (*SI Appendix*, Table S7). The fixed sample was imaged and quantified as described in the *SI Appendix, Material and Methods*. In these experiments, we measured cells that either survived (see *SI Appendix, Material and Methods* for criteria used to select surviving cells) or underwent “complete death” (*SI Appendix*, Fig. S3*A* and Movies S3 and S4). We identified complete death as the ceasing of most active processes on the cell surface and within the cell cytoplasm, a condition of “cellular stillness”.

### Caspase Dynamics Score Calculation.

The caspase dynamics score was calculated for each cell as follows: 1) cGC3AI maximal, time derivative maximal, and AUC were normalized as a fraction of their highest value, 2) values obtained in 1 were weighed according to the predictive power of the parameter consider (multiplied by the corresponding Tjur’s R^2^), and 3) a given cell was assigned a score calculated as the sum of its three weighed values.

### Stress Induction.

For short term, highly stressful confocal imaging 200,000 caspaseLOV cells were plated the day before the experiment on a glass-bottom 35 mm dish (Mattek #NC9903355). Imaging was carried out in 2 mL imaging media ±75 ng/mL DOX. After 3 h, a single focal plane was imaged and illuminated with blue light every 20 s for 50 min in a given position, before switching to a different position for three consecutive rounds of imaging. Detailed imaging and quantification procedures are reported in the *SI Appendix, Material and Methods*.

To induce proteotoxic stress, cells were treated with 75 ng/mL DOX±500 nM MG-132 (Millipore Sigma #474791). Imaging began 30 min post-treatment at 10 min intervals. The sample was washed after 3 h and imaging continued overnight. Detailed imaging and quantification procedures are reported in the *SI Appendix, Material and Methods*.

## Supplementary Material

Appendix 01 (PDF)

Movie S1. Dividing cGC3AI^+^ cell. Note the multiple rounds of pre-mitotic shrinkage. mCherry is shown in red, cGC3AI is shown in green in the merged panel and in gray. Time is shown in hh:mm. Related to Figure 3B.

Movie S2. Dividing cGC3AI^+^ cell. Note how the two daughter cells undergo transient shrinkage post-mitosis. mCherry is shown in red, cGC3AI is shown in green in the merged panel and in gray. Time is shown in hh:mm. Related to Figure 3C.

Movie S3. cGC3AI^+^ cell undergoing apoptosis until complete loss of membrane and cytoplasm movement. mCherry is shown in red, cGC3AI is shown in green. Time is shown in hh:mm. Related to Supplementary Figure S3A.

Movie S4. cGC3AI^+^ cell undergoing apoptosis after mitotic entry until complete loss of membrane and cytoplasm movement. mCherry is shown in red, cGC3AI is shown in green. Time is shown in hh:mm. Related to Supplementary Figure S3A.

Movie S5. cGC3AI activation (in green) during highly stressful live imaging. Related to Figure 6E.

## Data Availability

All study data are included in the article and/or *SI Appendix*.
