# Supplementary material for: Cell survival following direct executioner-caspase activation
Source: Proc Natl Acad Sci U S A. 2023 Jan 20;120(4):e2216531120. doi: 10.1073/pnas.2216531120 (PMC9942801; doi:10.1073/pnas.2216531120)
Supplement: Supplementary file 1 — Appendix 01 (PDF) [file pnas.2216531120.sapp.pdf]

Supporting Information for
Cell survival following direct executioner-caspase activation

Maddalena Nano[a,b,1], James A. Mondo[a], Jacob Harwood[a], Varuzhan Balasanyan[a,2], and Denise J. Montell[a,b,1]

Denise J. Montell
Email: dmontell@ucsb.edu

Maddalena Nano
Email: mnano@ucsb.edu

This PDF file includes:

Supporting list of non-standard abbreviations
Figures S1 to S9
Tables S1 to S8
Legends for Movies S1 to S5
Supporting Material and Methods
SI References

Other supporting materials for this manuscript include the following:

Movies S1 to S5

List of non-standard abbreviations

AUC	Area Under the Curve
C.I.	Confidence Interval
cC3	Cleaved caspase-3
cGC3AI	Cleaved GC3AI
DOX	Doxycycline
F.I.	Fluorescent Intensity
GC3AI	GFP-based caspase-3-like protease activity indicator
IntDen	Integrated Density
MGV	Mean Gray Value
MOMP	Mitochondria Outer Membrane Permeabilization
px	Pixel
Q-VD	Irreversible caspase inhibitor Q-VD-Oph
ROI	Region of Interest
SD	Standard Deviation
tGC3AI	Total GC3AI

SI - Figure S1 - Nano et al

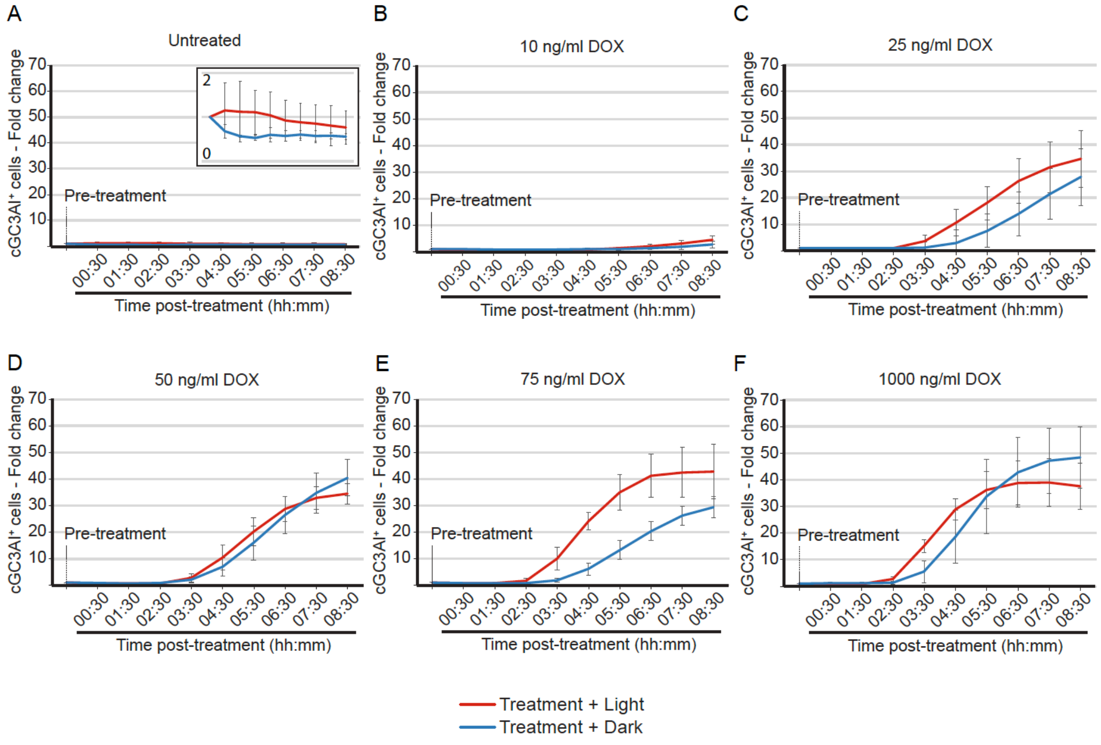

Supplementary Figure S1. Dynamics of GC3AI activation. A-F. Line charts indicating the normalized fold change in the number of GC3AI$^+$ cells over time in caspaseLOV expressing cells in untreated conditions (A) and in response to different DOX treatments (10 ng/ml in B, 25 ng/ml in C, 50 ng/ml in D, 75 ng/ml in E, and 1000 ng/ml in F) in the presence (red) or absence (blue) of illumination. GC3AI activation is almost absent at DOX concentrations up to 10 ng/mL (B). The first consistent response to DOX was observed at 25 ng/mL (C). Plotted values are from automated quantifications (n=3 independent experiments). Error bars=SD.

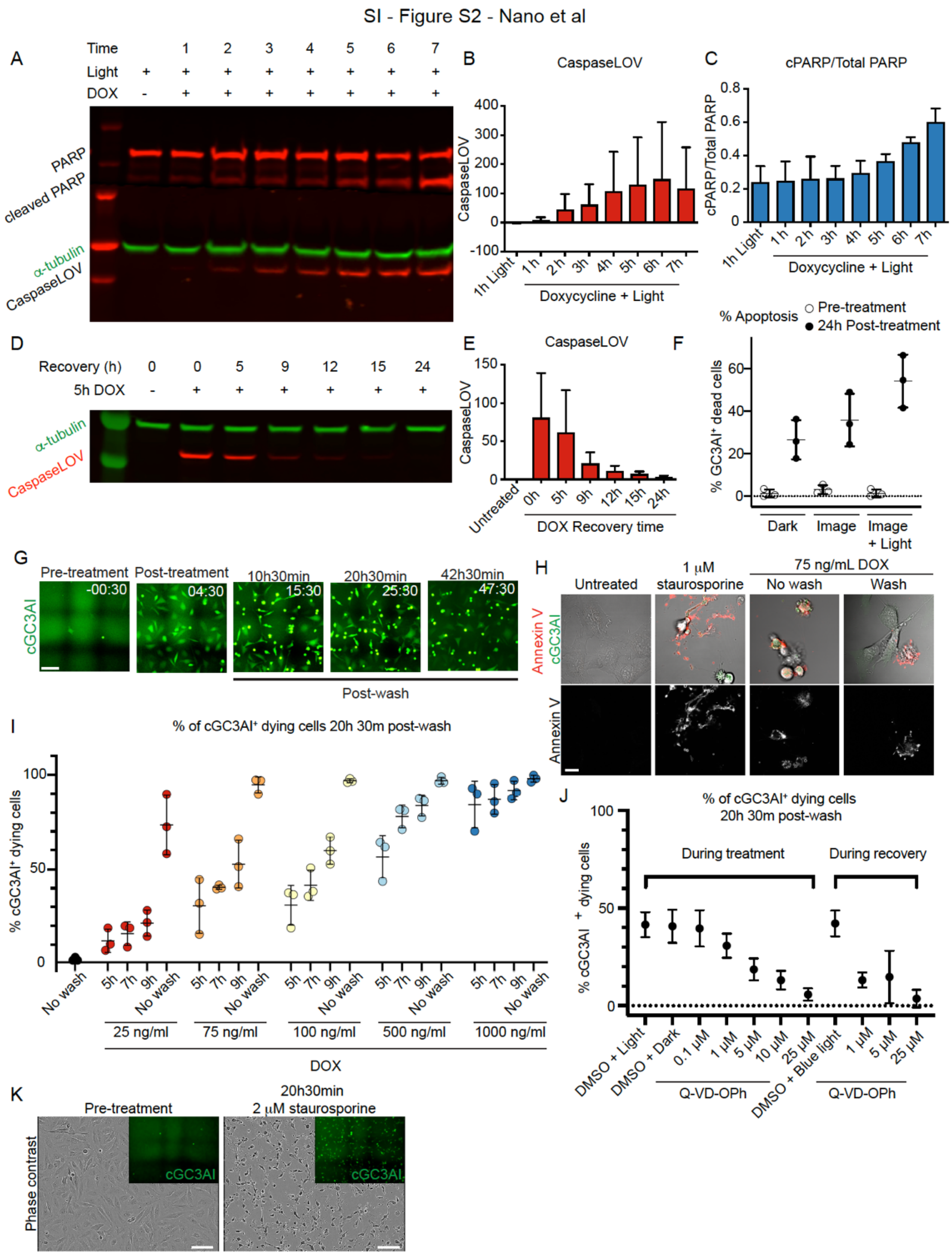

Supplementary Figure S2. Dynamics of CaspaseLOV activation and shutdown. A. Western blot with antibodies against PARP, caspase-3 (both in red) and α-tubulin as a loading control (in green,

50 kDa). The anti-caspase-3 antibody detects caspaseLOV (~40 kDa) at increasing concentrations as the incubation time with DOX increases (time is shown in h). PARP is detected at the expected molecular weight of 116 kDa. PARP cleavage over time is visible as an increase in the band located at lower molecular weights (cPARP, 89 kDa). **B-C.** Quantifications of A from 3 independent experiments. Error bars=SD. **D.** Western blot with antibodies against caspase-3 (in red) and α-tubulin as a loading control (in green, 50 kDa). CaspaseLOV levels decline over time during recovery. **E**. Quantifications relative to D from 3 independent experiments. Error bars=SD. **F.** Dot plot chart showing the percentage of cGC3AI^{+} dead cells pre-treatment (white dots) and 24h post-treatment (5h 75 ng/mL DOX followed by recovery)(black dots). Cells were kept in the dark, illuminated hourly only to acquire images or imaged and illuminated with blue light during the 5h of DOX incubation. Note how simple image acquisition is sufficient to increase the fraction of dying cells. **G.** Enhanced cGC3AI signal of stills presented in Figure 2D. Scale bar=100 μm. **H.** Annexin V staining (red) and cGC3AI (green) in response to different treatments. Annexin V binds phosphatidylserine when it is exposed on the cell surface during apoptosis. We detected Annexin V binding only at late apoptotic stages. In anastatic cells, binding was mostly undetectable. Scale bar=15 μm. **I-J.** Dot plots showing the percentage of dying cGC3AI^{+} cells in the population 20h 30min after DOX washout. Error bars=SD. n=3 independent experiments. **I.** Dot plot chart showing the percentage of dying cGC3AI^{+} cells treated with the indicated concentrations of DOX for the indicated time. **J.** Graph showing the average percentage of dying cGC3AI^{+} cells treated 5h with 75 ng/mL DOX and the indicated amount of Q-VD or mock (DMSO), added during treatment or during recovery. **K.** Stills of HeLa cells stably encoding the effector caspase sensor GC3AI (in green), mCherry and DOX-inducible caspaseLOV. Cells were not treated with DOX but were exposed to 2 μM staurosporine. Note how these cells are competent for apoptosis, evidenced by

GC3AI activation (insets) and cell shrinkage, despite lacking caspase-3 and caspaseLOV expression.

Scale bar=100 µm.

SI - Figure S3 - Nano et al

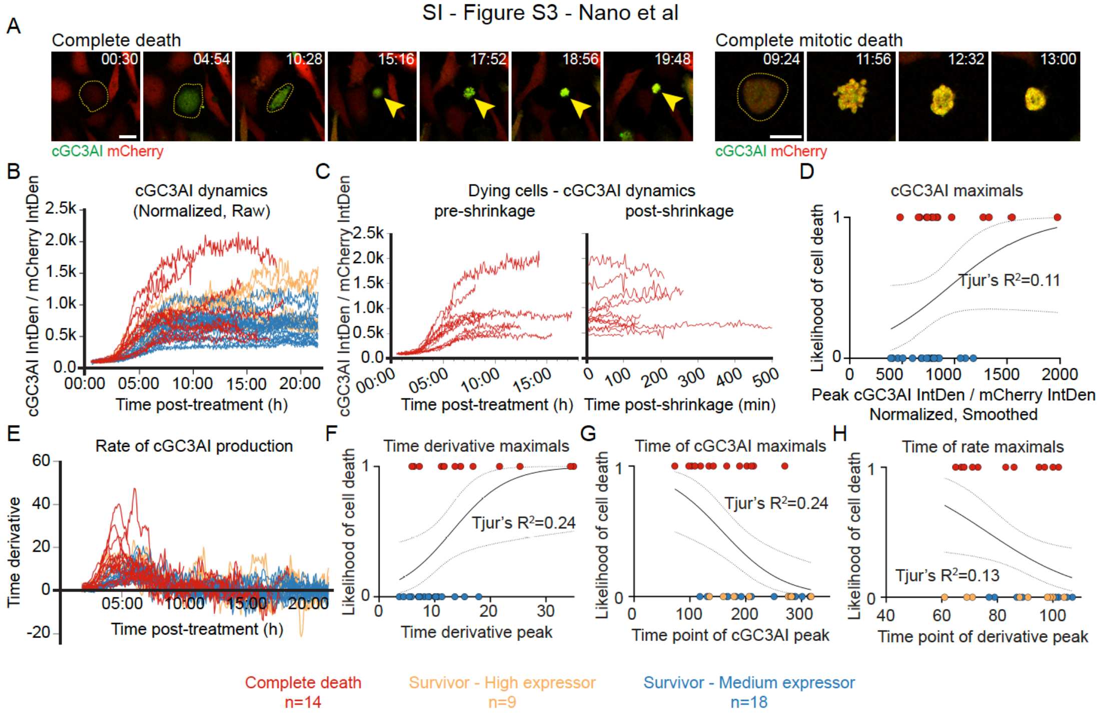

Supplementary Figure S3. Recovery plasticity from diverse cell death parameters. A. Stills of live imaging depicting cells undergoing “complete death”. Time is shown in hh:mm. Yellow dotted lines and arrowheads indicate the cell of interest. Scale bars=25 μm. Left panels: note how a blebbing phase (t=17:52) is followed by the emergence of cellular stillness, where most morphological active processes cease (from t=18:56). Right panels: apoptosis following mitotic entry. Note the darker cytoplasmic region at t=09:24, corresponding to the metaphase plate. After blebbing (t=11:56), the cell enters cytoplasmic stillness. **B.** Related to Figure 4A: line graph showing the dynamics of cGC3AI formation over time in dying (red) vs surviving (orange and blue) cells before smoothing. CaspaseLOV peak of expression occurs shortly after DOX washout (see Supplementary Figure S2D-E), explaining the plateauing of cGC3AI curves. **C.** Dynamics of cGC3AI formation over time in dying cells before and after shrinkage. **D.** Related to Figure 4B: logistic regression between the maximum fold change recorded for cGC3AI (denoted “maximal” or “peak”) and the likelihood of cell death (0=alive, 1=dead) for dying cells (red) and cells that express

medium levels of tGC3AI (blue). **E.** Related to Figure 4C: line graph showing the time derivative of curves in before smoothening. **F.** Related to Figure 4D: Logistic regression between the maximum of the time derivative (denoted “maximal” or “peak”) and the likelihood of cell death (0=alive, 1=dead) for dying cells (red) and cells that express medium levels of tGC3AI (blue). **G-H.** Logistic regression between the likelihood of cell death (0=alive, 1=dead) and the time at which cGC3AI (G) or the time derivative (H) reach their peaks. Tjur’s R^2 evaluates the goodness of fit. n=number of cells analyzed for each condition (from 3 independent experiments).

SI - Figure S4 - Nano et al

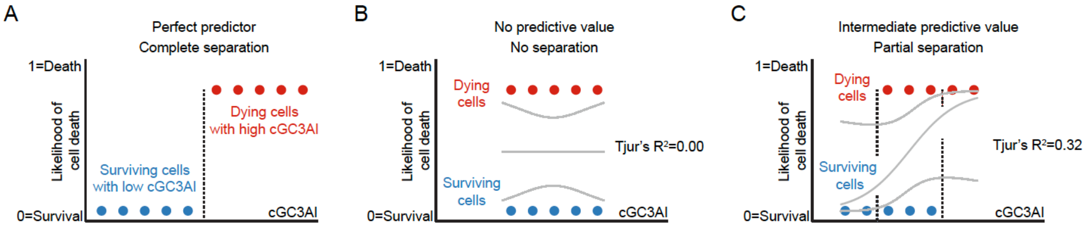

Supplementary Figure S4. Interpretation of logistic regression. A-C. Explanation of logistic regression. To fit a logistic regression, it is necessary to assign a binary value (0 or 1) to an event of interest. In our case, we assigned a value of 0 to cell survival, and a value of 1 to cell death. In this way, each cell could be placed in a graph according to its fate and the measured level of cGC3AI. **A.** cGC3AI is a perfect predictor of cell fate: all dying cells (red) have higher cGC3AI levels than surviving cells (blue). It is not possible to fit a logistic regression. cGC3AI levels perfectly separate dying from surviving cells. **B.** cGC3AI has no predictive value. Dying cells and surviving cells have overlapping values of cGC3AI. cGC3AI will give no indication as to whether the observed cell will die or survive, and the logistic regression will have a Tjur's R^2=0.00. The logistic regression and its 95% C.I. are shown in gray. **C.** cGC3AI has intermediate predictive value. In the example shown, dying cells and surviving cells have the same cGC3AI value in 3/10 cases. The logistic regression will have a Tjur's R^2=0.32. This means that a logistic regression model can fit (predict) about 30% of the observed outcomes.

SI - Figure S5 - Nano et al

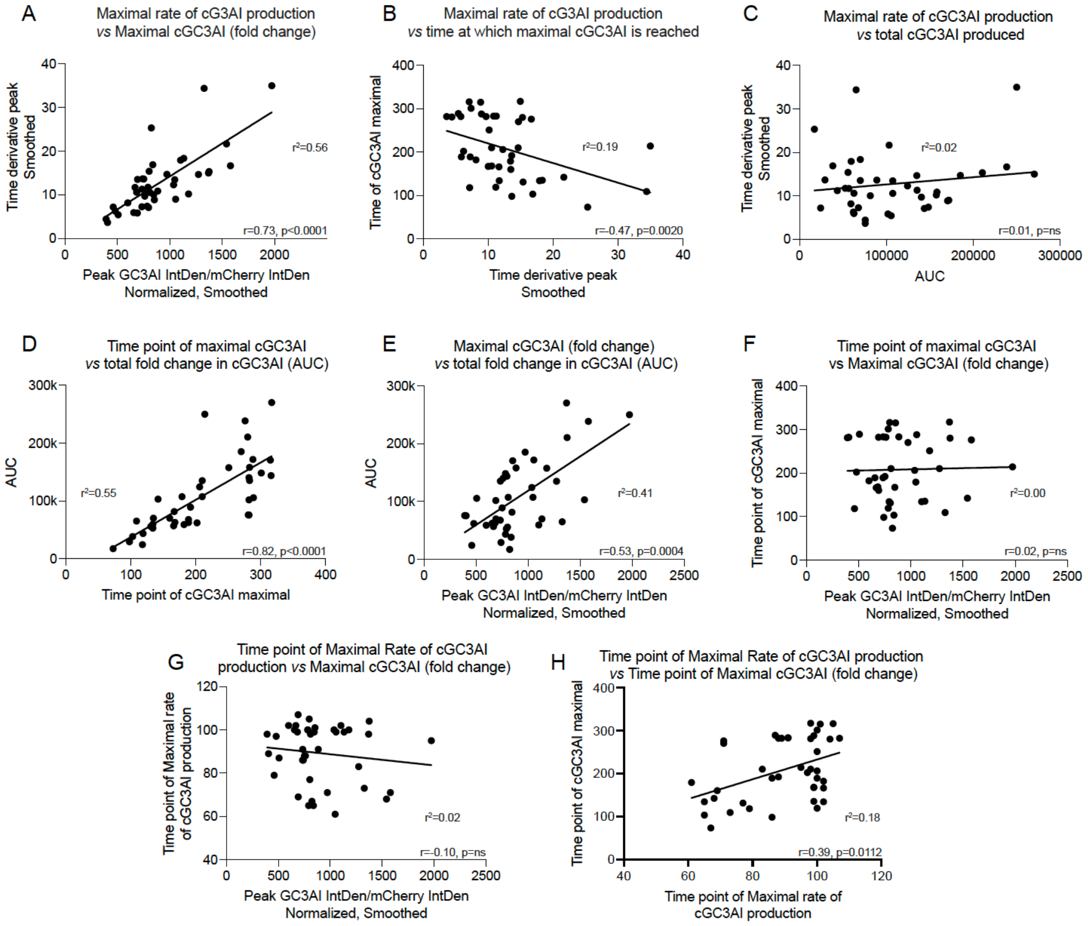

Supplementary Figure S5. Relationships between measures of cleaved caspase dynamics. A-H. Scatter plots and simple linear regressions between different parameters used to quantify caspase activity. r^2 indicates how well the linear regression fits the data. r (Spearman correlation coefficient) indicates whether data correlates (0=no correlation, 1=monotonic correlation). p=two-tailed P value. ns=non-significant. **A.** Correlation between maximal rate of cGC3AI production and maximal fold change in cGC3AI. Higher maximal rates of GC3AI cleavage correlate with higher cGC3AI peak levels. **B.** Correlation between maximal rate of cGC3AI production and time point at which maximal fold change in cGC3AI is reached. Each unit of time represents a 4min

window. Higher maximal rates of GC3AI cleavage correlate with earlier occurrence of cGC3AI peaks. **C.** Correlation between maximal rate of cGC3AI production and total cGC3AI accumulated (AUC=Area Under the Curve). High rates of cGC3AI production do not necessarily lead to accumulation of more total cGC3AI. **D.** Correlation between time point at which maximal fold change in cGC3AI is reached vs total cGC3AI accumulated (AUC). The duration of cGC3AI production contributes to the total amount of cGC3AI. **E.** Correlation between maximal fold change in cGC3AI and total cGC3AI accumulated (AUC). Large AUC values – which are determined by a combination of speed and duration of cGC3AI accumulation – are associated with higher cGC3AI peak level **F.** Correlation between maximal fold change in cGC3AI and time point at which it is reached. Higher maximal cGC3AI is not necessarily reached at earlier time points. **G.** Correlation between the time point at which the maximal rate of cGC3AI production is achieved and maximal fold change in cGC3AI. Production rates that peak earlier do not lead to higher maximal cGC3AI. **H.** Correlation between the time point at which the maximal rate of cGC3AI production is achieved and the time point at which the maximal fold change in cGC3AI occurs. Production rates that peak earlier anticipate cGC3AI peak occurrence.

SI - Figure S6 - Nano et al

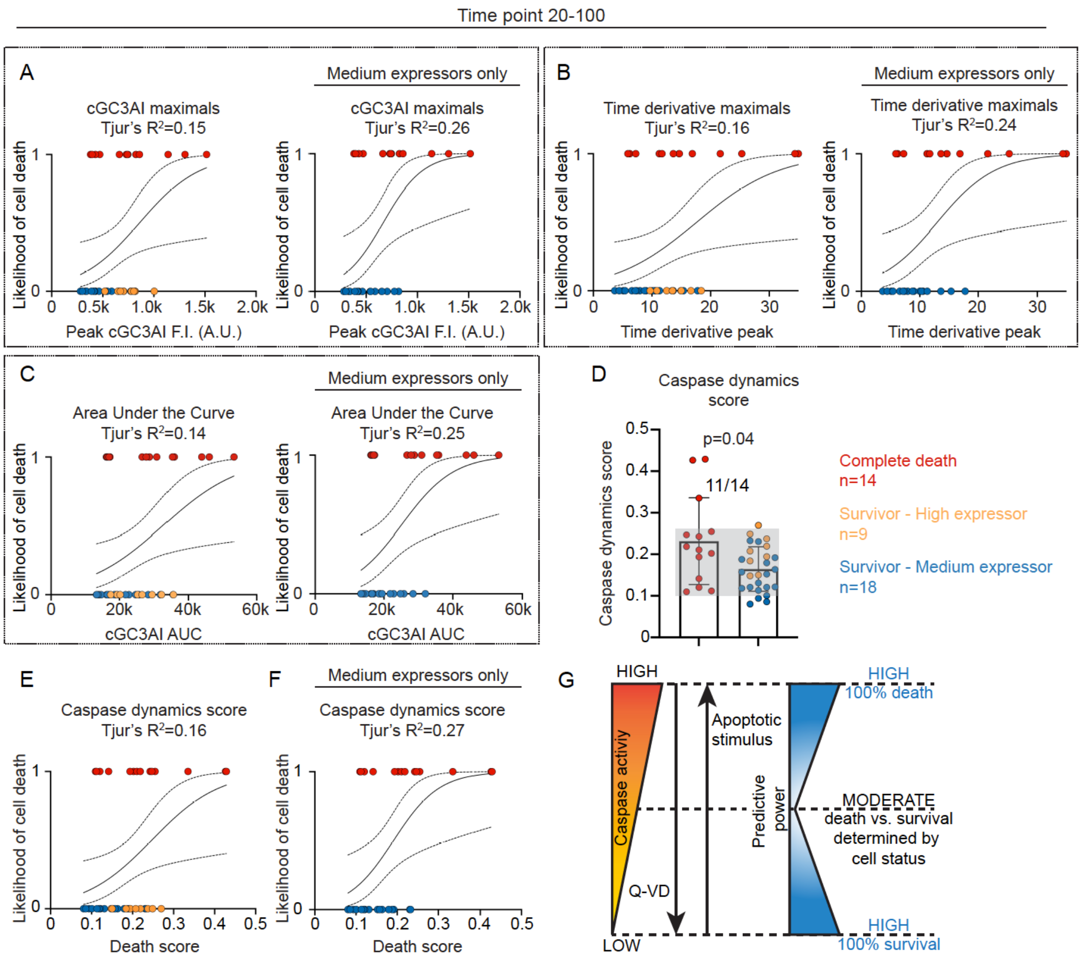

Supplementary Figure S6. Early GC3AI dynamics predictive power toward cell fate determination. A-C. Logistic regression between different parameters of cGC3AI generation during the first 100 time points of imaging (up to 428min post-treatment) and the likelihood of cell death (0=alive, 1=dead). Tjur's R^2 evaluates the goodness of fit. Dying cells are shown in red. Surviving cells are color-coded according to tGC3AI expression (blue=medium, orange=high). **A.** Related to Figure 4B and Supplementary Figure S3D: logistic regression between the maximum fold change recorded for cGC3AI (denoted "maximal" or "peak") and the likelihood of cell death. The predictive power of this parameter is higher when considering only its early values, which is consistent with dying cells cleaving GC3AI slightly faster than their surviving counterpart. **B.**

Related to Figure 4D and Supplementary Figure S3F: logistic regression between the maximum of the time derivative (denoted “maximal” or “peak”) and the likelihood of cell death. **C.** Related to Figure 4E: logistic regression between the AUC of curves in Figure 4A and the likelihood of cell death. Note how the logistic relationship is inverted when compared to Figure 4E. This can be explained with dying cells' tendency to accumulate cGC3AI faster at the beginning, together with the ability of surviving cells to accumulate cGC3AI for longer time intervals. **D-F.** Death score calculated considering only the first 100 time point of imaging. Related to Figure 5A-C. Dying cells are shown in red. Surviving cells are color-coded according to tGC3AI expression (blue=medium, orange=high). **D.** Related to Figure 5A. Dot plot chart and histogram showing the death score of dying and surviving cells. Error bars=SD. Statistical significance (p=0.04) was assessed with unpaired t test with Welch’s correction. Gray shading helps visualize dying and surviving cells with overlapping death scores. The numbers on top indicate the fraction of dying cells with death scores overlapping with surviving cells. **E-F.** Related to Figure 5B-C. Logistic regression between the death score calculated on the first 100 time point of imaging and the likelihood of cell death (0=alive, 1=dead) when considering all surviving cells (E) or medium expressors only (F). Dotted lines represent the 95% C.I.. Tjur’s R^2 evaluates the goodness of fit. n=number of cells analyzed for each condition (from 3 independent experiments). **G.** Model of caspase activity predictive power. High (red) and low (yellow) levels of caspase activity can predict cell death and survival with 100% accuracy (blue). Active caspase is inhibited by Q-VD in a dose-dependent manner, increasing the fraction of surviving cells. Stronger apoptotic stimulation increases the levels of active caspase, increasing the fraction of dying cells. Intermediate levels of caspase activity are permissive to cell survival and cell death and their predictive power is limited.

SI - Figure S7 - Nano et al

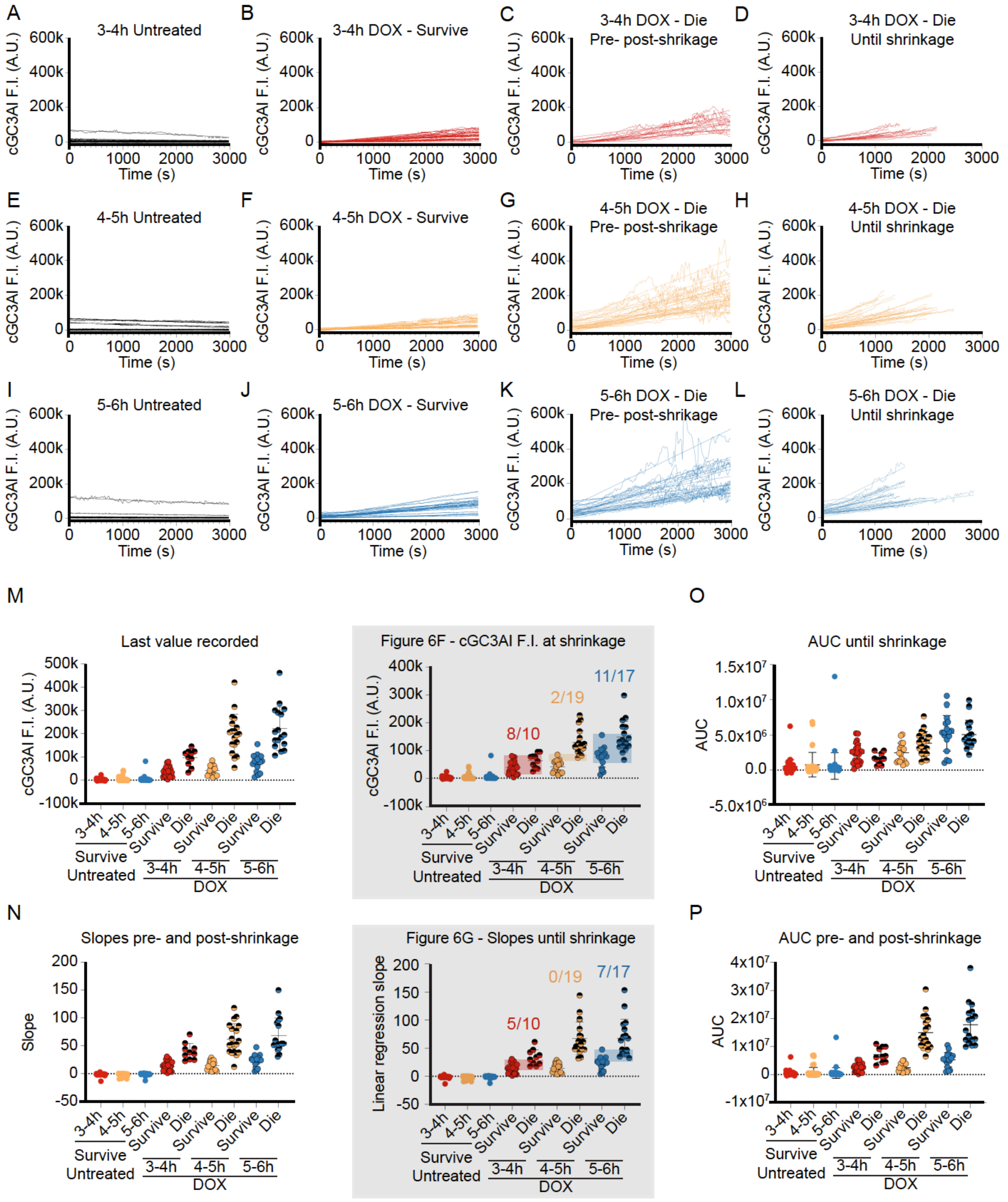

Supplementary Figure S7. Measurement of caspase activity beyond cell death overestimates the separation between dying and surviving cells. A-L. Line graphs and corresponding regression lines representing cGC3AI F.I. (A.U.) in untreated (A, E, I) and DOX-treated surviving (B, F, J) and dying (C-D, G-H, K-L) cells. Dying cells were measured for the whole duration of the imaging (C, G,

K) or until the moment of shrinkage (D, H, L). Data plotted are relative to the first (red), second (orange), or third (blue) hour of imaging. **M.** Dot plot showing the last value recorded for cGC3AI F.I. recorded at the end of imaging for dying and surviving cells (relative to curves A-C, E-G, I-K). Inset (light gray) re-proposes Figure 6F for comparison purposes, where measures stopped at the moment of death for dying cells (relative to curves A-B, D, E-F, H, I-J, L). Please note the difference in the y axis. Error bars=SD. **N.** Dot plot showing the slope of GC3AI fluorescence intensity over time from the beginning to the end of imaging for both dying and surviving cells (relative to curves A-C, E-G, I-K). Outlined inset re-proposes Figure 6G for comparison purposes, where measures stopped at the moment of death for dying cells (relative to curves A-B, D, E-F, H, I-J, L). Error bars=SD. **O-P.** Dot plots showing the AUC of dying and surviving cells. **O.** AUCs calculated from the beginning to the end of imaging for surviving cells, and until shrinkage for dying cells (relative to curves A-B, D, E-F, H, I-J, L). **P.** AUCs calculated from the beginning to the end of imaging (relative to curves A-C, E-G, I-K).

SI - Figure S8 - Nano et al

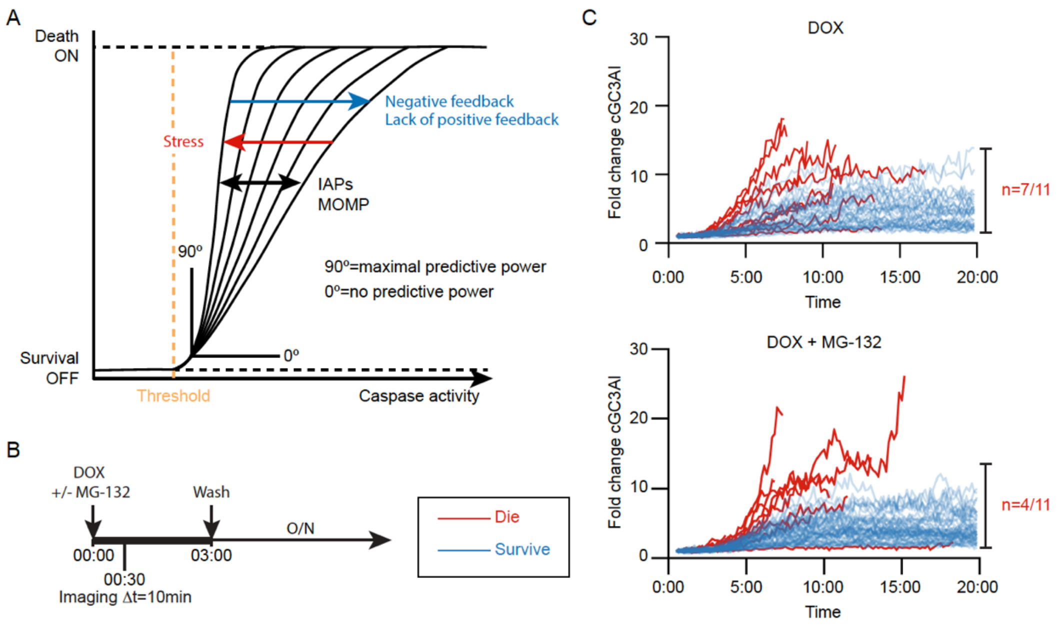

Supplementary Figure S8. The cell state contributes to death vs. survival choices. A. If reaching a threshold of caspase activity (orange dotted line) was sufficient to determine death vs. survival, the system (solid black lines) would transition immediately from the “off” to the “on” state (dotted black lines) and caspase activity would have maximal predictive power. In this scenario, the system would transition to the “on” state at a 90° angle. If caspase activity had no bearing on cell fate choices, the system state would not change with increasing caspase activity (0°). However, we show that the predictive power of caspase activity depends on the cell pre-existing state and can be increased by stress. Other mechanisms might be at play in determining how well caspase activity determines cell death, including negative feedback, lack of positive feedback, fluctuations in inhibitors of apoptosis (IAPs) and susceptibility to MOMP. **B.** Schematic representation of the experimental design used to induce caspase activation and proteotoxic stress. **C.** Line graphs of surviving (blue) and dying (red) cells treated with DOX±MG-132 from 3 independent experiments. MG-132 increases the separation of caspase activity curves between

dying and surviving cells. n=fraction of dying cells with levels of caspase overlapping with surviving cells. Time post-treatment is shown in hh:mm. Caspase activity levels are expressed as a fold change of cGC3AI from its value (RawIntDen/Area) at the beginning of imaging.

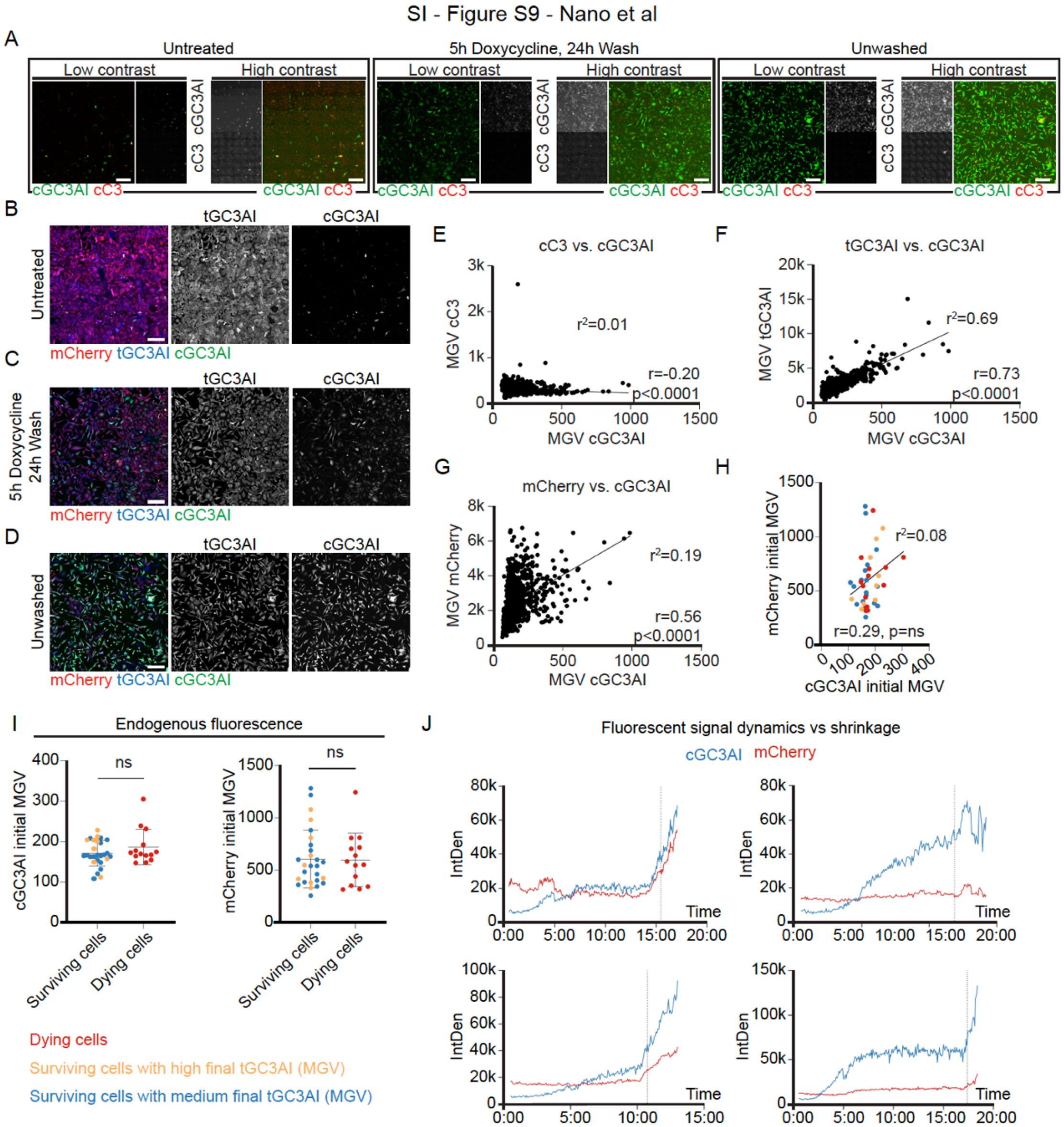

Supplementary Figure S9. Reliable cell sampling for quantification of GC3AI cleavage and dynamics. A-D. Validation of the immunofluorescence approach used in correlative live and fixed confocal microscopy. **A.** Micrographs of untreated cells and cells treated with 75 ng/mL DOX for 5h, with or without wash. Samples were stained with antibodies against cleaved caspase-3 (cC3, in red in the merged panel and in gray). cGC3AI was detected by endogenous green fluorescence (in green and in gray). The same images are shown with lower and higher contrast. cGC3AI sensitivity was much greater than obtained by immunostaining with antibody for cC3 (see

Supporting Material and Methods). Scale bar=200 μm. **B-D.** Same cells shown in A displaying endogenous mCherry (red), cGC3AI (green and gray), and stained with antibodies against GFP (blue and gray). The anti-GFP antibody recognizes both cleaved and uncleaved GC3AI (tGC3AI). Scale bar=200 μm **B.** Untreated cells. **C.** Cells treated 5h with 75 ng/mL DOX, washed and maintained in fresh media for 24h. Virtually all cells activated effector caspases (cGC3AI). **D.** Cells maintained in DOX for the whole duration of the experiment. **E-I.** Measurement of final (post-fixation, E-G) and initial (live, H-I) fluorescent markers in experiments of correlative live and fixed confocal microscopy. **E-G.** Scatter plots and simple linear regressions (r^2) between different fluorescent proteins after live imaging (post-fixation). r^2 indicates how well the linear regression fits the data. r (Spearman correlation coefficient) indicates whether data correlates (0=no correlation, 1=monotonic correlation). p=two-tailed P value. ns=non-significant. **E.** Poor correlation between cGC3AI detected by its endogenous fluorescence and cC3 (detected by antibody staining). This was expected, because cGC3AI sensitivity in detecting effector caspase activity is greater than cC3 (see Supporting Material and Methods). **F.** Correlation between cGC3AI detected by its endogenous fluorescence and tGC3AI (detected by antibody staining). **G.** Correlation between cGC3AI and mCherry, both detected by their endogenous fluorescence. Correlation was not expected and suggests that some cells behave as high expressors of exogenous proteins, and some as low expressors (see Supporting Material and Methods). **H.** Scatter plot and simple linear regression (r^2=0.08) between endogenous mCherry and cleaved GC3AI (cGC3AI) as measured at the beginning of live imaging (denoted "Initial cGC3AI"). MGV=Mean Gray Value. r=Spearman correlation coefficient. p=two-tailed P value. ns=non-significant. **I.** Dot plot charts showing initial cGC3AI and mCherry MGV in dying (red) vs surviving (orange and blue) cells. Lack of statistical significance was assessed with the Mann-Whitney test. Similar levels of initial mCherry in dying and surviving cells confirm that our sampling was unbiased

toward high or low expressors (see Supporting Material and Methods). **J.** Representative examples of cGC3AI and mCherry signal (IntDen) variation over time. Note that some cells experience a comparable increase in both signals when they shrink (likely due to water loss), while others do not.

Supplementary Table S1. Percentage of cells experiencing the indicated event in untreated (black) and DOX-treated (red) samples. n represent the number of cells scored for each condition. For cells that underwent mitosis, fate scoring was carried out as follows: if both daughter cells survived or died, that event was scored as one surviving or one dying cell; if one daughter died and one survived, that event was scored as one cell having "Opposite fates". We noted rare events of cell fusion (<1%), and examples of prolonged shrinkage followed by recovery of a flat morphology (~1%). >99% of cells did not undergo any fusion event, and ~99% of surviving cells maintained a normal morphology.

Condition	Surviving cells	Dying cells	Opposite fates	Mitosis	Cell fusion	Long shrinkage
Untreated<br>n=818	96.5%<br>n=789/818	3.3%<br>n=27/818	0.2%<br>n=2/818	44.3%<br>n=362/818	0.4%<br>n=3/818	0.0%<br>n=0/818
DOX<br>n=3328	75.0%<br>n=2495/3328	23.8%<br>n=791/3328	1.3%<br>n=42/3328	41.9%<br>n=1394/3328	0.5%<br>n=17/3328	1.1%<br>n=35/3328

Supplementary Table S2. Summary of the number of cells analyzed and their fate (relative to the experiment depicted in Figure 6A). Fluorescence measurements were only carried out for surviving and dying cells. Cells that divided (n=1) and cells that did not activate GC3AI in DOX-treated samples (n=5 cells in 3-4h and n=4 cells in 4-5h DOX treatment) are not reported. Black: untreated; red: 3-4h DOX; orange: 4-5h DOX; blue: 5-6h DOX.

Condition	Surviving cells	Dying cells	Unclassified
Untreated n=142	99.3% n=141/142	0.0% n=0/142	0.7% n=1/142
3-4h DOX n=44	52.3% n=23/44	22.7% n=10/44	25.0% n=11/44
4-5h DOX n=46	39.1% n=18/46	41.3% n=19/46	19.6% n=9/46
5-6h DOX n=48	37.5% n=18/48	35.4% n=17/48	27.1% n=13/48

Supplementary Table S3. Initial vs final average value of cGC3AI fluorescence intensity (in A.U.) for each condition tested and corresponding fold change. Relative to Figure 6B-D. Red: 3-4h; orange: 4-5h; blue: 5-6h.

Condition	Initial cGC3AI	Final cGC3AI	Fold change
3-4h untreated	6708.1	2037.5	0.3X
4-5h untreated	8576.8	4060.9	0.5X
5-6h untreated	6025.5	3483.1	0.6X
3-4h DOX survive	3575.0	37448.7	10X
4-5h DOX survive	5220.0	41341.1	8X
5-6h DOX survive	15144.6	76005.7	5X
3-4h DOX die	8115.3	96792.9	12X
4-5h DOX die	34034.0	199248.1	6X
5-6h DOX die	51530.9	222217.9	4X

Supplementary Table S4. CRISPR reagents.

ThermoFisher #A35509	Synthetic crRNA for CASP3<br><br>Target DNA sequence: GGAAGCGAATCAATGGACTC
ThermoFisher #A35506	TrueGuide™ tracrRNA
ThermoFisher #CMAX00001	Lipofectamine™ CRISPRMAX™ Cas9 Transfection Reagent
ThermoFisher #A36497	TrueCut™ Cas9 Protein v2

Supplementary Table S5. Cloning primers. (F)=forward primer, (R)=reverse primer.

Sequence (5'-3')	Template
(F) cgtcagatcgcctggagaattggccaccatgggttcttctcacc	LIC1B_Caspase-LOVN7C-7
(R) gagaagttcgtggctccggattagtgataaaaatagagttcttttgtgagcatggaaac	LIC1B_Caspase-LOVN7C-7
(F) gtttccatgctcacaaaagaactctatttttatcactaatccggagccacgaacttctc	pCW57-MCS1-2A-MCS2
(R) ggtgagaagaacccatggtggccaattctccaggcgatctgacg	pCW57-MCS1-2A-MCS2

Supplementary Table S6. List of plasmids.

Plasmid	Origin	Reference	Purpose
pCDH-puro-CMV-GC3AI	pCDH-puro-CMV-GC3AI was a gift from Binghui Li (Laboratory of Cancer Cell Biology, Key Laboratory of Breast Cancer Prevention and Therapy, Tianjin Medical University Cancer Institute and Hospital, Tianjin, China)	(1)	Lentiviral expression vector expressing puromycin resistance gene and a dark green sensor that fluoresces upon caspase cleavage under the control of CMV promoter (mammalian expression)
LIC1B_Caspase-LOVN7C-7	LIC1B_Caspase-LOVN7C-7 was a gift from Jim Wells (Addgene plasmid # 104629; http://n2t.net/addgene:104629; RRID:Addgene_104629)	(2)	Light-activated caspase-3 (bacterial expression)
pCW57-MCS1-2A-MCS2	pCW57-MCS1-2A-MCS2 was a gift from Adam Karpf (Addgene plasmid # 71782 ; http://n2t.net/addgene:71782 ; RRID:Addgene_71782)	(3)	DOX-inducible lentiviral expression vector (mammalian expression). Carries puromycin resistance
pCW57_CaspaseLOV	Nano M<br>Montell DJ	Current work	DOX-inducible lentiviral expression vector containing light-activated caspase-3 from LIC1B_Caspase-LOVN7C-7 (mammalian expression). Carries puromycin resistance
pCDH-CMV-mCherry-T2A-Puro	pCDH-CMV-mCherry-T2A-Puro was a gift from Kazuhiro Oka (Addgene plasmid # 72264 ; http://n2t.net/addgene:72264 ; RRID:Addgene_72264)	(4)	Expresses mCherry and puromycin resistance under the control of CMV promoter
pCMV-VSV-G	pCMV-VSV-G was a gift from Bob Weinberg (Addgene plasmid # 8454; http://n2t.net/addgene:8454; RRID:Addgene_8454)	(5)	Lentiviral packaging
pCMV-dvpr-dR8.2	pCMV-dR8.2 dvpr was a gift from Bob Weinberg (Addgene plasmid # 8455; http://n2t.net/addgene:8455; RRID:Addgene_8455)	(5)	Lentiviral packaging

Supplementary Table S7. Primary and secondary antibodies used in immunofluorescence.

Antibody	Raised in	Reference	Working concentration
anti-Phospho-Histone H2A.X (S139)	Mouse	Abcam #ab26350	1:100
Anti-Tom20	Mouse	Santa Cruz Biotechnologies #sc-17764	1:1000
anti-GFP	Chicken	Abcam #ab13970	1:1000
anti-cleaved Caspase 3 (Asp175) (D3E9)	Rabbit	Cell Signaling Technologies #9579	1:200
anti-Mouse IgG (H+L) Cross-Adsorbed Secondary Antibody, Alexa Fluor™ 647	Goat	Thermo Fisher Scientific #A-21235	1:1000
anti-Rabbit IgG (H+L) Cross-Adsorbed Secondary Antibody, Alexa Fluor™ 405	Goat	Thermo Fisher Scientific #A-31556	1:500
anti-Chicken IgY (H+L) Secondary Antibody, Alexa Fluor™ 647	Goat	Thermo Fisher Scientific #A-21449	1:1000
anti-Rabbit IgG (H+L) Cross-Adsorbed Secondary Antibody, Alexa Fluor™ 405	Goat	Thermo Fisher Scientific #A-31556	1:1000

Supplementary Table S8. Percentage of cells experiencing the indicated event in untreated (black) and DOX-treated samples. DOX-treatment occurred in the presence of illumination with the 458nm laser line at 0% (red), 5% (orange) or 10% (blue) laser power. Untreated samples were treated at 10% intensity to control for phototoxicity. For cells that underwent mitosis, fate scoring was carried out as follows: if both daughter cells survived or died, that event was scored as one surviving or one dying cell; if one daughter died and one survived, that event was scored as one cell having "Opposite fates".

Condition	Surviving cells	Dying cells	Opposite fates	Mitosis	Cell fusion	Long shrinkage
Untreated<br>n=818	96.5%<br>n=789/818	3.3%<br>n=27/818	0.2%<br>n=2/818	44.3%<br>n=362/818	0.4%<br>n=3/818	0.0%<br>n=0/818
Doxycycline - 0%<br>n=929	83.4%<br>n=775/929	15.7%<br>n=146/929	0.9%<br>n=8/929	47.9%<br>n=445/929	0.8%<br>n=7/929	1.1%<br>n=10/929
Doxycycline - 5%<br>n=1404	68.7%<br>n=965/1404	29.2%<br>n=410/1404	2.1%<br>n=29/1404	42.1%<br>n=591/1404	0.3%<br>n=4/1404	1.0%<br>n=14/1404
Doxycycline -10%<br>n=995	75.9%<br>n=755/995	23.6%<br>n=235/995	0.5%<br>n=5/995	36.0%<br>n=358/995	0.6%<br>n=6/995	1.1%<br>n=11/995

Movie S1 (separate file). Dividing cGC3AI^{+} cell. Note the multiple rounds of pre-mitotic shrinkage. mCherry is shown in red, cGC3AI is shown in green in the merged panel and in gray. Time is shown in hh:mm. Related to Figure 3B.

Movie S2 (separate file). Dividing cGC3AI^{+} cell. Note how the two daughter cells undergo transient shrinkage post-mitosis. mCherry is shown in red, cGC3AI is shown in green in the merged panel and in gray. Time is shown in hh:mm. Related to Figure 3C.

Movie S3 (separate file). cGC3AI^{+} cell undergoing apoptosis until complete loss of membrane and cytoplasm movement. mCherry is shown in red, cGC3AI is shown in green. Time is shown in hh:mm. Related to Supplementary Figure S3A.

Movie S4 (separate file). cGC3AI^{+} cell undergoing apoptosis after mitotic entry until complete loss of membrane and cytoplasm movement. mCherry is shown in red, cGC3AI is shown in green. Time is shown in hh:mm. Related to Supplementary Figure S3A.

Movie S5 (separate file). cGC3AI activation (in green) during highly stressful live imaging. Related to Figure 6E.

Supporting Material and Methods

Generation of caspaseLOV cells

HeLa cells (ATCC® CCL-2™) stably transduced with pCDH_GC3AI were subject to monoclonal selection by serial dilution. pCDH_GC3AI was a gift from Binghui Li (1) (Laboratory of Cancer Cell Biology, Key Laboratory of Breast Cancer Prevention and Therapy, Tianjin Medical University Cancer Institute and Hospital, Tianjin, China). The cell line obtained was modified by CRISPR-Cas9 to remove the endogenous gene encoding executioner-caspases-3. Caspase-3 was targeted using a crRNA:tracrRNA duplex guiding Cas9 (Supplementary Table S4). A monoclonal cell line was obtained by serial dilution and verified by sequencing (Eurofins) and western blot, using an anti-caspase-3 antibody (GeneTex #GTX110543). The cell line was then transduced with a DOX-inducible lentiviral plasmid for a light-sensitive caspase (Caspase-LOV). CaspaseLOV was amplified from LIC1B_Caspase-LOVN7C-7 (AddGene #104629)(2) and cloned in pCW57-MCS1-2A-MCS2 (3) (AddGene #71782) by overlap PCR (primers are listed in Supplementary Table S5). The resulting plasmid was verified by sequencing (Eurofin). A monoclonal cell line was obtained after transduction by serial dilution.

When indicated, caspaseLOV cells were further transduced with pCDH-CMV-mCherry-T2A-Puro. mCherry^{+} cells were sorted on a Sony SH800 Cell Sorter based on gating for mCherry positivity designed on the parental cell line. After gating for doublets and debris exclusion, 66.09% of the new cell population expressed mCherry.

Plasmids (listed in Table S6) were amplified in STBL3 bacterial cells (ThermoFisher #C737303). PCRs were carried out using PrimeSTAR® GXL DNA Polymerase (Takara #R050A), according to manufacturer instructions.

Lentivirus packaging was obtained using pCMV-VSV-G (AddGene #8454) and pCMV-dvpr-dR8.2 (AddGene #8455) (5). Hek 293-T cells were transfected with X-tremeGENE™ HP DNA Transfection Reagent (Sigma # 6366236001) and used for lentivirus production. The media containing lentiviral particles was collected 3 days post-transfection, spun down 2min at 2000rpm (RT) and filtered (0.45 µm Acrodisc® Syringe Filters PALL 4654; VWR #28143-352). The filtered media was used to transduce recipient cells in the

presence of 2 μg/mL polybrene (Sigma #1003). Recipient cells were centrifuged 30min at 2000rpm (RT) to improve transduction efficiency.

Blue light illumination

Blue light illumination for western blotting, IncuCyte imaging and immunofluorescence to detect DNA damage was achieved using a custom blue LED plate at 5V, 0.09-0.08A. During confocal imaging, acquisitions and illumination were ran on a Zeiss LSM 780 microscope with temperature and CO_2 control operated by Zeiss Black. Illumination during long term confocal imaging (Figure 3, Supplementary Table S8) was achieved scanning the sample every 4min with the 458nm laser during the first 4h 30min of imaging at 0%, 5% and 10% laser power (see also section "Long term confocal imaging"). For short-term, highly stressful confocal imaging (Figure 6, Supplementary Figure S7) bleaching was carried out at every acquisition (Δt=20sec) on the whole field of view (20 iterations, 458nm and 488 laser lines, 5% laser power). Note that the illumination used to image the green channel was likely sufficient to at least partially activate caspaseLOV, even in the absence of blue light.

IncuCyte imaging and image analysis

Cells were imaged using a Nikon 20X objective and filter module Dual Color Model 4459. Green (acquisition time=400msec) and phase contrast images were collected hourly. After a pre-treatment acquisition, imaging was resumed 30min post-treatment. Image size: 1392x1040 at 0.61 μm/pixel (px). Automated identification of green cells was achieved with the built-in analysis job function of the IncuCyte Zoom software that was calibrated with the following parameters: adaptive thresholding, GCU=0.5, Edge split=ON, Edge sensitivity=0 and minimum area=100. For manual counting, cGC3AI$^+$ cells (identified by eyes or automatically) were scored as alive when they had a flat morphology, and as dead when they had a round morphology (less than 5% of round cGC3AI$^+$ cells were mitotic). Automated quantifications are reported in Supplementary Figure S1 as a fold change in the number of cGC3AI$^+$ cells over time. Normalized data from independent experiments were averaged and plotted.

Western blotting and blots quantification

After collection, samples were re-suspended in 50 μL fractionation buffer (250 mM Sucrose (Sigma #84097), 20 mM HEPES (Thermo Fisher Scientific #15630080), 10 mM KCl (Sigma #409316), 1.5 mM $MgCl_2$ (Sigma #M1028), 1 mM EDTA (Sigma #E7889), 1 mM EGTA (BioWorld #40520008), 1 mM DTT (Sigma #43816), cOmplete™ EDTA-free Protease Inhibitor Cocktail (Sigma #4693132001)). Samples were then lysed in Laemmli sample buffer (Bio-Rad #1610747), boiled 15min at 90°C and run in 4-20% Mini-PROTEAN TGX precast protein gels (Bio-Rad #4561096). The primary antibodies used were mouse anti-α-Tubulin as loading control (1:3000, T6199; Sigma), rabbit anti-caspase-3 (1:1000, GeneTex #GTX110543), rabbit anti-PARP (46D11) (1:1000, Cell Signaling #9532). Secondary antibodies (used 1:5000) were IRDye® 800CW Goat anti-Mouse IgG (Li-Cor #926-32210) and IRDye® 680LT Donkey anti-Rabbit IgG (Li-Cor #926-68023). Blots were scanned using an Odyssey imaging system (LI-COR Biosciences). Experiments were run in triplicate. Representative blots are shown in the figures. Raw images were exported from Image Studio™ (LI-COR Biosciences), and relative quantification was made using Fiji (7).

For quantification, rectangular regions of interest (ROIs) of constant size were designed for a given protein and were used to measure the band fluorescent signal and the lane background, which was then subtracted from the band signal. The lane normalization factor was obtained dividing the observed α-Tubulin signal for a given lane by its highest value recorded. caspaseLOV signal was normalized using the lane normalization factor. Full-length PARP (FL-PARP) has an expected MW of 113 kDa, while its largest cleaved fragment (cPARP) has an expected weight of 89 kDa (8). We measured the bands located at approximately those molecular weights and did not consider additional bands. After normalizing both signals to the lane normalization factor, we calculated and plotted cPARP/Total PARP (Total PARP = cPARP + FL-PARP).

Immunofluorescence

After fixation, sample processing was carried out at room temperature (RT), protecting the sample from direct light to preserve endogenous fluorescence. The sample was rinsed in PBS and permeabilized 5min in PBS 0.1% Triton X-100 (Sigma #T8787). After 3x5min washes in PBS, the sample was blocked 20min in blocking buffer (PBS 0.1% Triton X-100, 1% bovine serum albumin (Sigma #A9647), 5% normal goat serum (Abcam #ab7481). It was then incubated with primary antibodies in blocking solution overnight at 4°C or 1h

RT in a humid chamber. After 3x5min washes in PBS, the sample was incubated with secondary antibodies ±DAPI (1:1000) in blocking solution 30min RT. The sample was washed 3x5min in PBS and was moved in fresh PBS until imaging. Alternatively, it was rinsed with Milli-Q water and mounted on a glass slide with VECTASHIELD® Antifade Mounting Medium (Vector Laboratories #H-1000-10).

Mitochondria abundance, activity, DNA damage and Annexin V assays

To determine mitochondria abundance and activity, we carried out correlative live and fixed confocal microscopy (n=3 independent experiments). 150.000 caspaseLOV-expressing cells were plated the day before the experiment on a glass-bottom 35 mm Dish (Mattek #NC9903355). The following day, cells were moved in imaging media with 10 nM Tetramethylrhodamine methyl ester perchlorate (TMRM, Millipore Sigma #T5428) ±75 ng/mL DOX, and were allowed to equilibrate 30min at the microscope before the beginning of imaging. 5h post-treatment, the sample was washed with 3 mL of fresh media with 10 nM TMRM. Imaging resumed immediately and continued O/N. Imaging media was prepared using DMEM, high glucose, HEPES, no phenol red (Thermo Fisher Scientific #21063029), and was supplemented with 10% heat-inactivated fetal bovine serum (Sigma-Aldrich #F4135) and 50 units/mL Penicillin-Streptomycin (Gibco #15070063). TMRM is a potentiometric, cation dye, that accumulates in active mitochondria.

Live Imaging was carried out on a Zeiss LSM 780 laser scanning confocal microscope with temperature and CO_2 control operated by Zeiss Black a Plan-Apochromat 20x/0.8 M27 air objective. A single focal plane was acquired. Large image size to prevent cell migration outside the field of view (x: 1275.29 µm, y: 1275.29 µm) was achieved by Tile scanning (3x3 tiles). Dimensions: x: 3073, y: 3072, time: 67 time points during treatment + 250 time points post-treatment (Δt=240sec), 16-bit. Px dwell: 0.64 µsec. Average: line, 4. Green and red fluorescence acquisitions were set up on two different tracks. GC3AI was imaged with the 488 nm laser line (10% laser power, 800V master gain, detected on ChS2 with 490-562 filter, pinhole: 31.6 µm=1 A.U.=1.8 µm section; beam splitters used: MBS 488/561/633, MBS -405). TMRM and brightfield DIC were imaged with the 561 nm laser line (3% laser power, 750V and 200V master gain, detected on ChS2 with

597-696 filter and ChD respectively, pinhole: 38.1 µm=1 A.U.=2.2 µm section; beam splitters used: MBS 488/561/633, MBS -405).

After live imaging, the sample was acquired one more time. It was then immediately fixed and stained as described, using an anti-Tom20 antibody to identify mitochondria, detected with a secondary antibody conjugated to Alexa Fluor 405 (Supplementary Table S7). The fixed sample was imaged on the same microscope using a Plan-Apochromat 40x/1.4 Oil DIC M27 (dimensions: 16 bit, 7168x7168px, 1487.84μm^2; px dwell=0.64µsec lasers: 5% 488nm, 5% 633nm, 20% 405nm, 10% 561nm; detection: ChS1 491-571nm 800V, Ch2 651-755nm 680V, Ch1 410-483nm 850V, ChS2 571-651nm 800V, and ChD 150V).

A random selection of $GC3AI^-$ untreated cells and $GC3AI^+$ DOX-treated cells were quantified. Fixed images were used to design a binary mask of the mitochondrial network based on the Tom20 signal after despeckling, gaussian blurring (σ=2) and constant thresholding. The mask was further processed using the distance transform watershed command (parameters used: Chessboard: 1,1; Normalized; Dynamic=4.0; Connectivity=4). The network identified by the mask was used to measure the area of the mitochondrial network. It was then manually aligned to the pre-fixation image and used to measure TMRM fluorescence intensity (Mean Gray Value) after background subtraction (rolling ball radius: 100px).

For evaluation of DNA damage 33.000 caspaseLOV cells/well were plated in 24 well plates on top of sterilized 12mm circular glass coverslips (Fisher Scientific #12-545-80P). Cells were treated 5h with media ±75 ng/mL DOX in the presence of blue light illumination. After 5h, cells were washed with fresh media and allowed to recover 24h without illumination before fixation and immunostaining. DNA double strand breaks were detected using an antibody recognizing Phospho-Histone H2A.X (S139), detected with a secondary antibody conjugated to Alexa Fluor 647 (Supplementary Table S7). Samples were stained with DAPI to visualize the DNA. Data collected are from n=2 independent experiments. For image quantification, we designed a nuclear mask using the DAPI signal, and we used it to measure DNA damage in the nuclear region in single cells (DNA damage was expressed as Raw IntDen/Area). The background was calculated manually as the average signal (Raw IntDen/Area) of ROIs of 19x19px placed in the cytoplasm of every cell in the field

of view and was subtracted to the DNA damage signal. Imaging was carried out on a Zeiss LSM 800 operated by Zeiss Blue using a Plan-Apochromat 40x/1.2 Imm Korr DIC M27 objective. Phospho-Histone H2A.X conjugated to a far-red secondary antibody (excitation: 653nm, 20% laser power) was detected at 656-700nm on a GaAsP-Pmt2 detector (700V). Pinhole: 35μm. DAPI (excitation: 353nm, 3% laser power) was detected at 410-546nm on a GaAsP-Pmt1 detector (550V). A single focal plane was acquired. Dimensions:1024x1024px, 159.73μm^2, bit depth:16 bit, px time: 1.03 μsec.

Phosphatidylserine exposure on apoptotic cells was detected by Annexin V Red Dye binding (Sartorious #4641). Cells were plated in 8 well chamber μ-Slides (Ibidi #80827). Once cells reached approximately 60% confluency, they were treated with media±1μM staurosporine or media±75ng/mL DOX. DOX-treated cells were either washed after 5h or kept in DOX. The day after treatment, the media was changed to imaging media 1:200 Annexin V Red Dye. The living sample was imaged at the Zeiss LSM 780 microscope using 1% 561nm laser power (detected on transmission detector with 300V master gain and on ChS1 410-696 with 750V master gain and pinhole 1.01A.U.=0.8um section, MBS488/561/633 and MBS-405) and 3% 488nm laser power (detected on ChS2 490-562, master gain: 750V; pinhole=1.01 A.U.=0.8um section; MBS 488). The two different lasers were acquired on separate tracks. We used a 40X Plan-Apochromat 40x/1.4 Oil DIC M27 objective to image a single focal plane. Dimensions: x: 1024, y: 1024, px size=0.21 μm, bit depth=16-bit, Px dwell: 0.64 μsec. Average: line, 4.

Long term confocal imaging

Imaging media was prepared as described above. To prevent thermal drift and allow for sample stabilization, the sample was placed on the microscope incubation stage and allowed to rest for 30min before the beginning of imaging. Imaging was carried out on a Zeiss LSM 780 laser scanning confocal microscope with temperature and CO_2 control operated by Zeiss Black using a Plan-Apochromat 20x/0.8 M27 air objective. A single focal plane was acquired. Large image size to prevent cell migration outside the

field of view (x: 1274.74 µm, y: 1274.87 µm) was achieved by Tile scanning (3x3 tiles). Dimensions: x: 3073, y: 3072, time: 67 time points during treatment + 233 time points post-treatment (Δt=240sec), channels: 4 (during treatment) and 3 (post-treatment), 16-bit. Px dwell: 0.64 µsec. Average: line, 4. GC3AI was imaged with the 488nm laser line (10% laser power, 800V master gain, detected on ChS2 with 490-562 filter, pinhole: 31.6 µm=1 A.U.=1.8 µm section; beam splitters used: MBS 488/561/633, MBS -405). mCherry and brightfield DIC were imaged with the 561 nm laser line (1% laser power, 650V and 300V master gain, detected on ChS2 with 597-696 filter and ChD respectively, pinhole: 38.1µm=1A.U.=2.2 µm section; beam splitters used: MBS 488/561/633, MBS -405). The 458 nm laser line was used to deliver blue light illumination during the first 4h 30min of imaging (at 0%, 5% and 10% laser power, 500V master gain, detected on ChS1 with 410-598 filter, pinhole: 30 µm; beam splitter used: MBS 458/561). Note that it is possible that CaspaseLOV reached complete activation in our imaging system just from the 488-light delivered to image GC3AI, and further illumination might contribute to cell death independently from direct caspase activation. Image analysis was run in Fiji as described in the section "Image quantification and fate scoring for long term confocal imaging".

Image quantification and fate scoring for long term confocal imaging

For evaluation of cell fate, calculation of cell survival, mitotic rate and cell fusion rate (Supplementary Table S1 and Supplementary Table S8), all cells that did not disappear from the field of view were quantified from beginning to end of imaging (300 time points). If one cell divided and both daughter cells survived, that event was scored as one surviving cell. If one cell divided and one daughter died and one survived, that event was scored as one cell having "Opposite fates". If one cell divided and both daughter cells died, that event was scored as one dying cell. All cells that were not entirely shrunk by the end of imaging, were scored as surviving. The total number of cells is given by the sum of dying cells, surviving cells and cells with opposite fates. Cell fusion was considered to occur every time two cells collapsed into one. Mitotic failure post-furrowing was scored as daughter cell fusion.

For fluorescence intensity measurements in sister cells (Figure 3D), we designed 1 square ROI (15x15px) in the cell cytoplasm and changed its position to ensure it remained in the cell for the whole time

of interest. For each ROI, we measured the Integrated Density (IntDen) of cGC3AI and mCherry signals and calculated their ratio (IntDen cGC3AI/IntDen mCherry). The ratio was then expressed as a fold change of its value at time point 2 (equaled to 100) and was re-normalized post-washout.

For cGC3AI measurements in sister cells with opposite fates (Figure 3E), both sisters' mean fluorescence intensities were measured at the moment in which the dying cell shrunk. We normalized cGC3AI signal over mCherry and calculated the ratio of signal intensity between dying and surviving sisters. A ratio greater than 1 represents higher caspase activity in the dying sister, a ratio of 1 represents equal activity between sisters, and a ratio lower than 1 represents higher caspase activity in the surviving sister.

Correlative live and fixed confocal microscopy: validation of the immunofluorescence approach

Using caspaseLOV cells expressing mCherry, we analyzed tGC3AI expression levels (which includes the uncleaved, dark and the and the cleaved, fluorescent fractions) and its correlation with cGC3AI, cleaved caspase-3 (cC3) and the unrelated mCherry protein (Supplementary Figure S9A-D). To evaluate tGC3AI expression we used an anti-GFP antibody (Supplementary Table S7), and we found that it successfully stained cells even in the absence of active caspase (Supplementary Figure S9B-D), indicating that it could detect tGC3AI. In addition, cGC3AI sensitivity was much greater than obtained by immunostaining with antibody for cC3, which only convincingly stained a handful of cells (Supplementary Figure S9A). This was expected, since cGC3AI can be produced by any DEDVdase (1), and every active caspase molecule can amplify the signal cleaving multiple GC3AI molecules. Fixed confocal microscopy (relative to Supplementary Figure S9A-D) was carried out on a Zeiss LSM 780 laser scanning confocal microscope operated by Zeiss Black using a Plan-Apochromat 40x/1.4 Oil DIC M27 objective. 10 focal planes at 2 µm intervals were acquired. Fixed images were acquired with a frame size of 1024x1024 (212.5x212.5µm). Scanning: tile (7x7 tiles, 7168x7168px, 1487x1487µm), frame. Px size=0.21 µm, px dwell=0.64 µsec, bit depth=16-bit. Average=8 (line, mean). Green and far-red fluorescence acquisitions were set up on one single track (pinhole=0.7 µm section) and blue and red fluorescence acquisitions were set up on a different track (pinhole=0.7 µm). Laser lines and detection: 405nm (10%, Ch1 - 750V, filter: 410-483), 561nm (3%, ChS2 –

650V, filter: 571-651), 488nm (7%, ChS1 – 650V, filter: 491-571), 633nm (5%, Ch2 – 700V, filter: 651-755). Brightfield DIC was imaged with the first track and detected on ChD (150V).

Correlative live and fixed confocal microscopy and data analysis

Live imaging settings: Imaging settings were adjusted to ensure that signal did not reach saturation even in the most dramatic instances of cell shrinkage. Live Imaging was carried out on a Zeiss LSM 780 laser scanning confocal microscope with temperature and CO_2 control operated by Zeiss Black on a Plan-Apochromat 20x/0.8 M27 air objective. A single focal plane was acquired. Large image size to prevent cell migration outside the field of view (x: 1275.29 µm, y: 1275.29 µm) was achieved by Tile scanning (3x3 tiles). Dimensions: x: 3073, y: 3072, time: 67 time points during treatment + 250 time points post-treatment (Δt=240sec), 16-bit. Px dwell: 0.64 µsec. Average: line, 4. Green and red fluorescence acquisitions were set up on two different tracks. GC3AI was imaged with the 488 nm laser line (3% laser power, 700V master gain, detected on ChS2 with 490-562 filter, pinhole: 30.0 µm=0.96 A.U.=1.8 µm section; beam splitters used: MBS 488/561/633, MBS -405). mCherry and brightfield DIC were imaged with the 561 nm laser line (0.5% laser power, 550V and 300V master gain, detected on ChS2 with 597 - 696 filter and ChD respectively, pinhole: 30.0 µm=0.78 A.U.=1.8 µm section; beam splitters used: MBS 488/561/633, MBS -405).

Imaging post-fixation settings: we acquired 10 focal planes at 2 µm intervals. A field of view of 1.49 mm^2 was imaged by Tile scanning (7x7 tiles). Dimensions: x: 7167, y: 7168, 16-bit. Average: line, 8. Green and far red fluorescence acquisitions were setup on one single track (pinhole: 26.9 µm=0.85 A.U.=0.7 µm), blue and red fluorescence acquisitions on a different track (pinhole: 26.9 µm=1.01 A.U.=0.7 µm). Laser lines and detection: 405 nm (10%, Ch1 - 750V, filter: 410-483), 561 nm (3%, ChS2 – 650V, filter: 571-651), 488 nm (7%, ChS1 – 650V, filter: 491-571), 633 nm (5%, Ch2 – 700V, filter: 651-755). Brightfield DIC was imaged with the first track and detected on ChD (150V).

Fixed images quantifications. Fixed image measurements were made in Fiji after background subtraction (rolling ball radius=150px). The intensities measured in fixed imaging are referred to as "final" values and

are available only for surviving cells, because dead cells were lost during sample processing. The Mean Gray Value (MGV) of total GC3AI (tGC3AI) was used to select the surviving cells quantified in the live imaging (see section “Live imaging quantifications”). Image processing began by cropping the image so that it would roughly correspond to the last live frame (to facilitate cell identification between live and fixed images). For cell identification, we built a binary mask on a maximum projection of all z slices for the mCherry signal. The image was despeckled, blurred (gaussian blur, σ=2), converted to 8-bit and a reasonable threshold was applied to include most of the cell cytoplasm while maintaining individual cells separation. We applied distance transform watershed processing to the binary mask (parameters: chessboard 1,1; 32 bits; Normalized weights; Dynamic=10; Connectivity=4). After processing, each cell mask was manually added to the ROI manager using the magic wand tool. Touching cells were separated by manual mask correction. For fluorescence intensity measurements of endogenous GFP (cGC3AI), endogenous mCherry, tGC3AI and cC3, we measured Mean Gray Values (MGVs) on a single z-slice (if all tiles were in focus) or on a maximum projection of a few slices. Results are presented in Supplementary Figure S9E-G. We found that the MGV of cGC3AI and cC3 did not correlate, which is consistent with low cC3 sensitivity (Supplementary Figure S9E). cGC3AI and tGC3AI MGVs were highly positively correlated (Supplementary Figure S9F). This indicates that having more tGC3AI leads to the accumulation of more cGC3AI. Surprisingly, cGC3AI and mCherry were also moderately correlated (Supplementary Figure S9G). This suggests that some cells behave as high expressors of both mCherry and tGC3AI, while others behave as low expressors.

Live imaging quantifications. To exclude the possibility that some of the cells that we scored as dead might recover, we restricted our analysis to those that underwent complete loss of membrane and cytoplasm movement (Supplementary Figure S3A, Movies 3-4). First, we identified cells that underwent “complete death” and the moment in which they underwent shrinkage. If death occurred after mitotic entry, we defined shrinkage as the first time point in which morphological abnormalities (such as blebbing) became evident. For each movie, we also measured 9 surviving cells, that were selected according to their level of tGC3AI (as measured by immunofluorescence in the fixed sample). To control for heterogeneities in tGC3AI expression, we measured 6 surviving cells with near-average levels of tGC3AI (3 above-average and 3 below-

average), and 3 cells expressing high tGC3AI (selected amongst the highest expressors). Image measurements were run in Fiji after background subtraction (rolling ball radius=200px). We excluded: 1) cells that we could not match between fixed and live imaging, 2) cells that were not visible for the whole duration of the experiment, 3) cells that had high levels of cGC3AI from the beginning of imaging (higher than the majority of cells in the field of view), 4) cells for which cGC3AI values did not double by DOX washout, and 4) cells that were affected by imaging artifacts (tile transition affecting brightness, change in focal plane, overlap to other cells).

Quantifications of green and red fluorescence were carried out by manually designing 1 square ROI (15x15px) in the cell cytoplasm and changing its position to ensure it remained in the cell for the whole time of interest. Live imaging quantifications are presented in Figure 4, Figure 5, Supplementary Figure S3B-H, Supplementary Figure S5, Supplementary Figure S6.

To start, we tracked the selected surviving and dying cells back to the beginning of imaging, and we measured the initial MGV of cGC3AI and mCherry. We found that initial cGC3AI and mCherry were not significantly correlated (Supplementary Figure S9H). This is contrast with what we observed for the final, fixed values (Supplementary Figure S9G), and suggest that the moderate correlation observed for cGC3AI and mCherry final values only occurs when the majority of tGC3AI has been cleaved. Importantly, when we measured initial cGC3AI and mCherry, we found that they were not significantly different between dying and surviving cells (Supplementary Figure S9I), indicating that our approach successfully sampled subpopulations of low and high expressors.

For measuring the dynamics of caspase activation over time, we calculated the ratio between cGC3AI and mCherry signals (IntDen cGC3AI/IntDen mCherry) and normalized it as follows: 1) for each cell, we calculated the IntDen Ratio; 2) for each time point measured, we expressed the ratio as a fold change of its value at time point 2 (equaled to 100); 3) we re-normalized post-washout values expressing them as a fold change of the first time point after washout, which was equaled to the value of the last pre-washout time point. Normalized data are presented in Supplementary Figure S3B-C.

For calculating the parameters of caspase dynamics, we smoothed data using a moving average with interval=20 (Figure 4A). We then calculated the maximal value of the curves (peak; Figure 4B and

Supplementary Figure S3D), its time (Supplementary Figure S3G), the Area Under the Curve (after manually cropping data at the time point of cGC3AI maximal; Figure 4E) and the time derivative (Supplementary Figure S3E), using a custom Python code (available upon request) and manual interventions. Time derivatives maxima and their timing were obtained after further smoothing (Figure 4C-D, Supplementary Figure S3F, H). To determine the predictive role of caspase activity toward cell fate determination, we ran a logistic regression between different parameters of caspase dynamics and assigned a score of 0 to surviving and a score of 1 to dying cells. Statistical analysis and plotting were run in GraphPad Prism Version 9.3.1 (350) for Mac (GraphPad Software, La Jolla California USA, www.graphpad.com).

Linear regression of data is presented in Supplementary Figure S5. Data analysis limited to the first 100 time points of imaging is shown in Supplementary Figure S6.

Stress induction

Short term, highly stressful confocal imaging and image analysis. Imaging was carried out on a Zeiss LSM 780 laser scanning confocal microscope with temperature and CO_2 control operated by Zeiss Black using a Plan-Apochromat 40x/1.4 Oil DIC M27 oil objective. Bleaching was carried out at every acquisition (Δt=20sec) on the whole field of view (20 iterations, 458 nm and 488 nm laser lines, 5% laser power). Live imaging started 3h post-DOX (note that no wash step was included in this set of experiments). Samples were imaged and illuminated with blue lights every 20sec for 50min in a given position (3-4h DOX) (Figure 6A and F, Movie 5). Imaging was repeated in a total of 3 different positions (3-4h, 4-5h and 5-6h post-DOX treatment)(Figure 6A). Untreated samples were imaged in the same way.

We acquired an image size of 212.34x212.34 µm. Dimensions: x: 1024, y: 1024, time: 150 (Δt=20sec), channels: 2, 16-bit. Px dwell: 0.64 µsec. Average: line, 4. The 488 nm and 561 nm laser lines used for imaging GC3AI and brightfield DIC (5% and 3% laser power, respectively) were detected on the ZEISS QUASAR multichannel photomultiplier ChS1 detector with 491 - 571 filter (master gain: 750V, pinhole: 32 µm) and on the transmitted light detector ChD (master gain: 210V). The pinhole was set to 1 A.U. (0.8 µm section). Beam splitter used: MBS 488/561.

Cells that divided (n=1), cells that had unclear fate (defined below and reported as "unclassified" in Supplementary Table S2) and cells that did not activate GC3AI in DOX-treated samples (n=5 cells in 3-4h and n=4 cells in 4-5h DOX treatment) were not quantified. Cells were scored as dead when they fully shrunk forming a round, blebbing unit. Cells were scored as surviving when they experienced no shrinkage or limited shrinkage, but they still maintained some flat and dynamic edges. Cells were assigned unclear fate when they underwent sudden and very relevant but incomplete shrinkage affecting all cell edges, not linked to migration events.

Image analysis was run in Fiji. Images were quantified by manually designing 1 to 3 square ROIs (15x15px) in the cell cytoplasm. When possible, ROIs were designed so that they would remain in the cell for the whole duration of imaging. When cells migrated outside the initial ROI, new ROIs were designed. We did not quantify cells that moved excessively, making ROI design too challenging, or cells that moved outside the field of view or the focal plane before the end of imaging. A similar strategy was used for measuring the background. For each ROI, we calculated the brightness of the green channel (Raw IntDen/ROI Area). We calculated the average brightness for the background and subtracted it to the brightness of each ROI for a given cell. Cells from all experiments were grouped according to treatment (untreated/DOX), time of treatment (3-4h, 4-5h, 5-6h post-treatment) and fate (death/survival). For representation of cGC3AI dynamics over time (Figure 6B-D), all values measured from the beginning to the end of imaging were averaged and plotted.

Caspase activity predictive power should be evaluated right before apoptotic commitment has occurred. Measurements run too early are likely to include values permissive to recovery. Measurements run too late are likely to overestimate the amount of caspase activity required for the commitment to occur. In our analysis of long-term confocal imaging and correlative live and fixed confocal microscopy, we noticed that some dying cells' fluorescence steeply rose after shrinkage. This was not always due to an increase in caspase activity, since it could be observed for both cGC3AI and mCherry (Supplementary Figure S9J), and was normalized by their ratio, but was likely caused by water loss and fluorescent protein concentration. In the absence of mCherry to control for this effect, we decided to stop our predictive analysis for short term confocal imaging at the moment of shrinkage for dying cells. Since shrinkage is a dynamic phenomenon that

can have different durations and dynamics, we identified “beginning of shrinkage” as the moment in which we could detect changes in cell volume affecting the nucleus (slight contraction or deformation) or important volume decrease if the nucleus was not visible. For predictive analysis, surviving cells were measured for the whole duration of imaging, while dying cells were measured until cell death (25 time points after the beginning of shrinkage)(Figure 6F-S). Measures relative to the entirety of imaging for both dying and surviving cells can be found in Supplementary Figure S7. In addition, in the absence of mCherry, cGC3AI fluorescence intensity underwent fluctuations in intensity that were not entirely normalized by background subtraction (mostly linked to slight shifts in the focal plane). Therefore, to estimate the rate of caspase activity in each cell, we did not use the time derivative, but we ran a linear regression and calculated its slope. Since cGC3AI curves did not plateau, the AUC used for predictive analysis was calculated until the end of imaging for surviving cells, and until the moment of shrinkage for dying cells (Figure 6P-S, Supplementary Figure S7O). To determine the predictive role of caspase activity toward cell fate determination, we ran a logistic regression between calculated values (either slope, AUC or cGC3AI brightness) and assigned a score of 0 to surviving and a score of 1 to dying cells. Statistical analysis and plotting were run in GraphPad Prism Version 9.3.1 (350) for Mac (GraphPad Software, La Jolla California USA, www.graphpad.com).

Proteotoxic stress induction and image analysis. Imaging of caspaseLOV-expressing cells was carried out on a Zeiss LSM 780 laser scanning confocal microscope with temperature and CO_2 control operated by Zeiss Black using a Plan-Apochromat 20x/0.8 M27 air objective. A single focal plane was acquired. Due to the sensitivity of the cell line to apoptotic stimulation combined to confocal imaging, treatment time was reduced to 3h, and images were acquired every 10min. Large image size to prevent cell migration outside the field of view (x: 1275.29 µm, y: 1275.29 µm) was achieved by Tile scanning (3x3 tiles). Dimensions: x: 3073, y: 3072, time: 15 time points during treatment + 102 time points post-treatment, 16-bit. Px dwell: 0.64 µsec. Average: line, 4. GC3AI was imaged with the 488nm laser line (3% laser power, 750V master gain, detected on ChS2 with 490-562 filter, pinhole: 31.6 µm=1 A.U.=1.8 µm section; beam splitters used: MBS 488/561/633, MBS -405). Brightfield DIC was imaged with the 561 nm laser line (0.5% laser power, 300V master gain, detected on ChD).

Cells that migrated outside the central tile of imaging (before shrinkage for dying cells), cells that became superimposed to other cells preventing accurate measurements, cells that did not complete mitosis, cells that had unclear fate, cells positive for cGC3AI from the beginning of imaging, and cells that did not double cGC3AI levels at the last time point acquired were not quantified.

Image analysis was run in Fiji. Images were quantified by manually designing 1 square ROIs (15x15px) in the center of the cell. Measurements of cGC3AI activation were made after background subtraction (rolling ball radius=140px). For each ROI, we calculated the brightness of the green channel (Raw IntDen/ROI Area), normalized it as a fold change of its initial value, and plotted it using GraphPad Prism Version 9.3.1 (350) for Mac (GraphPad Software, La Jolla California USA, www.graphpad.com).

For dying cells, we stopped our measurement 20min before shrinkage, to avoid the artifactual rise in fluorescent signal that can be triggered by morphological changes (Supplementary Figure S9J).

SI References

1. J. Zhang, *et al.*, Visualization of caspase-3-like activity in cells using a genetically encoded fluorescent biosensor activated by protein cleavage. *Nat. Commun.* **4**, 2157 (2013).

2. A. D. Smart, *et al.*, Engineering a light-activated caspase-3 for precise ablation of neurons in vivo. *Proc. Natl. Acad. Sci. U. S. A.* **114**, E8174–E8183 (2017).

3. C. J. Barger, C. Branick, L. Chee, A. R. Karpf, Pan-Cancer Analyses Reveal Genomic Features of FOXM1 Overexpression in Cancer. *Cancers* **11** (2019).

4. M. Inoue, *et al.*, Plasma redox imbalance caused by albumin oxidation promotes lung-predominant NETosis and pulmonary cancer metastasis. *Nat. Commun.* **9**, 5116 (2018).

5. S. A. Stewart, *et al.*, Lentivirus-delivered stable gene silencing by RNAi in primary cells. *RNA N. Y. N* **9**, 493–501 (2003).

6. Q. Liu, *et al.*, Bim escapes displacement by BH3-mimetic anti-cancer drugs by double-bolt locking both Bcl-XL and Bcl-2. *eLife* **8**, e37689 (2019).

7. J. Schindelin, *et al.*, Fiji: an open-source platform for biological-image analysis. *Nat. Methods* **9**, 676–682 (2012).

8. G. V. Chaitanya, A. J. Steven, P. P. Babu, PARP-1 cleavage fragments: signatures of cell-death proteases in neurodegeneration. *Cell Commun. Signal. CCS* **8**, 31 (2010).
